# Out-of-Plane Aptamer Functionalization of RNA Three-Helix Tiles

**DOI:** 10.3390/nano9040507

**Published:** 2019-04-02

**Authors:** Aradhana Chopra, Sandra Sagredo, Guido Grossi, Ebbe S. Andersen, Friedrich C. Simmel

**Affiliations:** 1 Physik-Department E14, Technische Universität München, 85748 Garching, Germany; aradhanachopra@gmail.com (A.C.); sandra.sagredo@tum.de (S.S.); 2 Interdisciplinary Nanoscience Center (iNANO), Aarhus University, 8000 Aarhus C, Denmark; ggrossi@inano.au.dk (G.G.); esa@inano.au.dk (E.S.A.)

**Keywords:** RNA nanotechnology, aptamers, cotranscriptional folding

## Abstract

Co-transcriptionally folding RNA nanostructures have great potential as biomolecular scaffolds, which can be used to organize small molecules or proteins into spatially ordered assemblies. Here, we develop an RNA tile composed of three parallel RNA double helices, which can associate into small hexagonal assemblies via kissing loop interactions between its two outer helices. The inner RNA helix is modified with an RNA motif found in the internal ribosome entry site (IRES) of the hepatitis C virus (HCV), which provides a 90° bend. This modification is used to functionalize the RNA structures with aptamers pointing perpendicularly away from the tile plane. We demonstrate modifications with the fluorogenic malachite green and Spinach aptamers as well with the protein-binding PP7 and streptavidin aptamers. The modified structures retain the ability to associate into larger assemblies, representing a step towards RNA hybrid nanostructures extending in three dimensions.

## 1. Introduction

Over the past two decades, RNA has been found to be involved in many essential cellular processes other than the conventional roles it fulfils as mRNA, tRNA, or rRNA. Non-coding RNAs (ncRNAs) and many RNA–protein complexes are involved in regulatory functions at the transcriptional and translational levels, and have roles in scaffolding [1], genome-editing, RNA interference, clustered regularly interspaced repeats (CRISPR), and chromatin remodeling [2]. Many naturally occurring ncRNAs fold and assemble into complex 3D architectures via a plethora of secondary and tertiary interactions, and also via association with a wide range of RNA-binding proteins. More recently, the exceptional folding capability and modularity of biological RNAs have inspired the emergence of RNA nanotechnology, which aims at the construction and assembly of artificial nanostructures made from RNA [3]. Compared to DNA, RNA offers a variety of interesting features as a material for nanotechnology: RNA nanostructures can draw from a diverse variety of naturally occurring tertiary motifs [4,5,6], they can be enzymatically generated in large amounts via transcription, and they can be genetically encoded and expressed in cells [7,8].

Most naturally occurring RNAs are single-stranded and contain self-complementary sequences that facilitate intramolecular folding into distinct secondary structures. In addition, rigid structural motifs consisting of canonical or noncanonical base-pairing, kissing interactions, and stacking of helices play a significant role in RNA folding, resulting in complex 3D structures exhibiting helices, loops, junctions, bulges, stems, hairpins, and pseudoknots [4,5,6]. Seeking inspiration from the design principles found in nature and employing naturally occurring RNA motifs, several assembly strategies have been developed for RNA nano-construction. They include RNA architectonics [9,10], self-assembly of RNA/DNA hybrids [11,12] and single-strand RNA assembly [13]. RNA architectonics is based on the modular character of RNA, which allows 3D RNA motifs to be organized in alternative combinations in order to create different RNA nano-architectures. Large libraries of thermodynamically stable modular RNAs, which include both structural and functional motifs, have been identified and characterized from natural RNA molecules. Furthermore, such motifs have been used as suitable “parts” for designing self-assembling RNA units (or tectoRNAs) [10]. For instance, in this context, in silico methods have been developed to screen for natural RNA motifs capable of self-assembling into closed ring structures [14]. The DNA/RNA hybrid strategy leverages the properties of both RNA and DNA. It has been used for producing large nucleic acid structures based on the DNA origami technique, where a large RNA scaffold is folded with a number of complementary DNA/RNA staples. Single strand RNA assembly relies on a number of RNA strands that are unstructured by themselves but when mixed together assemble into a structure [12].

A technique dubbed cotranscriptional ssRNA origami has been developed, in which a single RNA strand folds into a predefined RNA tile that further assembles into hexagonal and rectilinear lattices while the RNA is produced by the RNA polymerase [13]. It employs a variety of RNA tertiary motifs to mediate the intra- and inter-tile interactions. Improved unimolecular DNA and RNA folding strategies have been developed based on minimizing the knotting complexity by employing parallel crossovers to avoid kinetic trapping during folding. This has resulted in RNA nanostructures of a variety of shapes and up to 6000 nt in length [15].

Rationally designed RNA nanostructures and devices have great potential for applications in synthetic biology, metabolic engineering, and nanomedicine [3,16,17,18,19]. In particular, the three-way junction from pRNA (a component of the phage phi29 packaging motor) has been found to be an extremely stable motif that can be used as the basis of multifunctional RNA nanoparticles for therapeutic applications [19]. More recently, RNA tiles designed by the cotranscriptional ssRNA origami technique have been used in combination with fluorogenic RNA aptamers to function as nanoscale aptamer-based Förster resonance energy transfer (FRET) sensors [20]. Additionally, programmed folding of RNA nanostructures of different shapes has also been performed in vivo [21]. Furthermore, hybrid multicomponent RNA-protein nanostructures have been characterized in vivo and in cell-free gene expression systems [22].

In the present work, we construct a novel three-helix “antiparallel even” RNA tile (3H-AE) based on the cotranscriptional ssRNA origami approach. In our design, the outer two helices of the 3H-AE tile provide a rigid RNA scaffold that can interact with other tiles via kissing loop (KL) interactions. The central RNA double-helical extensions, however, are conceived as modular plug-in modules, which can be modified at will without interfering with two-dimensional tile assembly. Specifically, we modified the plug-in helices with subdomain IIa of the internal ribosomal entry site (IRES) of the Hepatitis C virus (HCV) genome [23], which allowed attachment of arbitrary RNA modules perpendicularly protruding either above or below the plane defined by the 3H-AE RNA tile. As examples, we positioned the two fluorogenic malachite green (MG) [24] and Spinach [25,26] aptamers above and below the tile plane, respectively, which allowed us to monitor functional assembly of the tile structures via fluorescence spectroscopy. In order to demonstrate the modularity of the design, the top (malachite green) aptamer was also replaced with other aptamers such as the PP7 aptamer for the viral coat protein PCP [27,28] and an RNA aptamer for streptavidin [22]. We finally show that the streptavidin aptamer module can be used to immobilize the RNA tiles on streptavidin coated surfaces, while presenting a second function via another aptamer, indicating the potential for functionalizing surfaces with unmodified proteins or other ligands in controlled orientations. 

Our results demonstrate the modularity of the RNA origami tile approach and represent a step towards multifunctional RNA assemblies extending in three dimensions. The most salient feature of the three-helix tile structure introduced in this work is the modular extension of the middle helix with aptamer functions. This allows for connecting the tiles in 2D while presenting separate binding modalities on the two sides of the 2D assembly. Furthermore, it is conceivable to create 3D lattices from such structures by also polymerizing along the *z*-direction.

## 2. Materials and Methods

### 2.1. Design of Three-Helix RNA Tiles (3H-AE)

**Figure 1:**
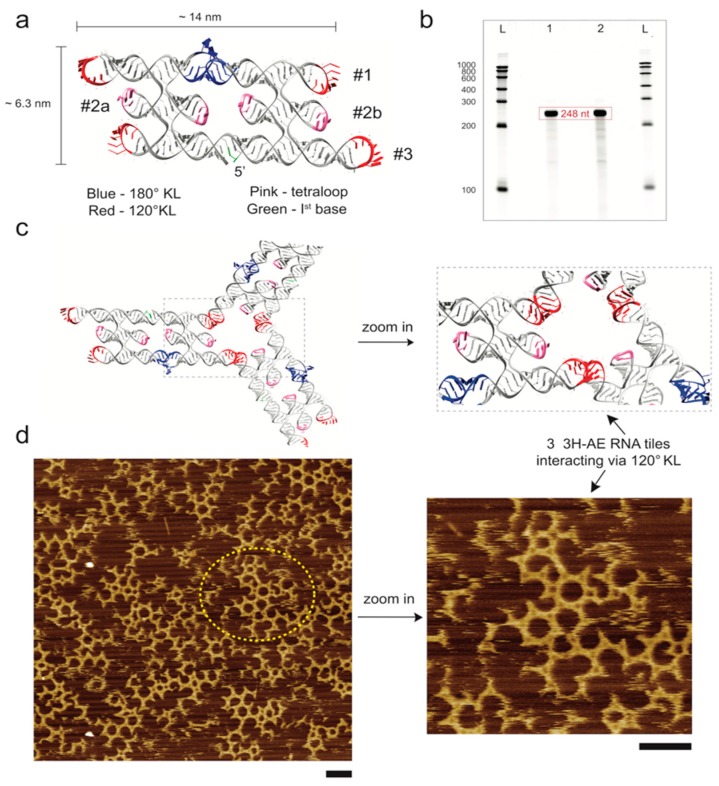
Design and characterization of the 3H-AE RNA tile. (**a**) schematic representation of designed 3H-AE RNA tile. Structural motifs are color coded as described in the legend; (**b**) denaturing PAGE gel showing the correct length of the RNA tile. L: LowRange RiboRuler, 1-2: RNA tile transcribed from DNA template amplified from Phusion High-Fidelity Master mix with HF or GC, buffer, respectively. (**c**) 3H-AE RNA tiles can interact with each other via 120° kissing loop (KL) motifs; (**d**) AFM images showing correct assembly and interaction of the RNA tiles. Tile assemblies were prepared by snap-cooling followed by incubation on mica at 37 °C (cf. Materials and Methods). The region enclosed by the dashed circle is further zoomed in to show the interactions of the 120° KL. Scale bars: 50 nm.

3H-AE and its subsequent modifications were designed by the cotranscriptional ssRNA origami method that has been previously described in detail [13,29]. Briefly, using the 3D modeling programs Swiss-PdbViewer [30] and UCSF Chimera [31], three standard A-form RNA double helices (#1-3, Figure 1a) were positioned over one another and rotated to create an optimal spacing for an anti-parallel even (AE) double crossover (DX). An internal 180° KL (HIV-1 DIS, PDB ID: 2B8R) [32] was placed between the crossovers on helix number 1 (top). UUCG tetraloops (extracted from PDB ID: 1F7Y) [33] were positioned at the ends and in between crossovers on helix number 2 (middle). In addition, 120° KLs (RNA i/ii inverse loop, PDB ID: 2BJ2) [34] were positioned at the ends of double helices numbers 1 and 3 to allow formation of hexagonal lattices. The 180° KL forms 6 base pairs between the two loops resulting in a coaxial stack and is in phase with the A-form helix of RNA, whereas the 120° KL forms 7 base pairs between the loops resulting in a continuous, but bent coaxial stack. Modifications in the 3H-AE design included additional RNA motifs such as subdomain IIa (PDB ID: 2PN4) and domain IIa (PDB ID: 1P5M) of the IRES of the HCV genome, which were used as connectors between the RNA tile and the malachite green aptamer (MGA) (PDB ID: 1F1T) [24]. These domains consist of a 90° angle [35,36] which allows for almost perpendicular arrangement of the added RNA motifs to the tile. An additional RNA sequence encoding Spinach aptamer (PDB ID: 4KZD) [26] was connected to 3H-AE with MGA V1 via subdomain IIa to generate the modified structure 3H-AE-MGA-Spinach. Additional variants of 3H-AE-MGA-Spinach without 120° KL were constructed. The MGA was further replaced by either a streptavidin aptamer [22] or an aptamer binding to bacteriophage PP7 coat protein fused with mCherry (PCP-mCherry, PDB ID: 2QUX) [28], respectively.

After the initial modeling, the 3D structures were ligated with a Perl script (“ligate.pl”, which was available from [37] and refined using a recursive geometric refinement function in the program *Assemble* [38]. 3D models in this work were rendered in UCSF Chimera. The designed structure was further traced using a Perl script (“trace.pl”, which is also available from [37]) and an input was generated that was used for the design of the corresponding RNA sequences in Nupack [39]. The sequences of the 180° KLs, 120° KLs, tetraloops, subdomain IIa, domain IIa, MGA, Spinach aptamer and PP7 aptamer were chosen from the PDB files and added to the respective designs (assuming a stable folding behavior of the aptamers, the corresponding sequences were modularly replaced, keeping the rest of the 3H-AE sequence constant). Additionally, some of the base pairs in the dovetail seam were constrained to be strongly stacking G-C pairs in an attempt to immobilize it to a static position. Sequences were further constrained to contain at least one G-U wobble pair per every eight continuous base pairs in order to avoid secondary structures in the RNA-encoding DNA template and simplify its synthesis. All of the remaining positions were designed by Nupack. The 5’-end of each sequence was constrained to begin with GGG, an optimal leader sequence for T7 RNA polymerase. In addition, 2D blueprints of the final structures resulting from this process are shown in Supplementary Figure S1.

Primers were generated specific to DNA sequence generated by Nupack and their melting temperature was calculated using the NEB Tm calculator [40].

### 2.2. Preparation of RNA Tiles

Genetic templates for all RNA tiles were amplified from “custom dsDNA gBlocks” from Integrated DNA Technologies (IDT) using the polymerase chain reaction (PCR). RNA tiles were then prepared using in vitro transcription from these templates as described in detail in the Supplementary Methods.

### 2.3. Characterization of RNA Tiles and Tile Assemblies

The formation of the RNA tiles was characterized using gel electrophoresis and atomic force microscopy (AFM). The transcription of RNA tiles modified with fluorogenic aptamers was followed using fluorescence spectroscopy. RNA tiles containing streptavidin aptamers were further investigated using streptavidin-coated microbeads. Detailed experimental procedures are found in the Supplementary Methods.

## 3. Results and Discussion

### 3.1. Design and Folding of the 3H-AE RNA Tile

We designed a novel three-helix “antiparallel even” (3H-AE) RNA tile as described in detail in the Methods section above. In contrast to previously described RNA tiles [13], it was constructed from *three* RNA double helices placed over one another connected via double crossovers, and converted into a cotranscriptionally folding continuous ssRNA using one 180° KLs, four 120° KLs and four tetraloops (Figure 1a). The central (inner) helices (#2a and #2b) of the 3H-AE tile were modularly functionalized with various aptamers as discussed in the following sections.

Custom dsDNA segments containing the sequence for the 3H-AE RNA tile and its subsequent modifications were ordered from a gene synthesis supplier and PCR amplified using a Phusion High-Fidelity PCR Master mix (with HF or GC buffer). The purity and amplification of the samples was checked using agarose gel electrophoresis (Supplementary Figure S2). Transcription and folding of RNA structures (3H-AE RNA tile, 248 nt) were verified via a denaturing PAGE. The transcribed RNA was observed at the correct length as shown in Figure 1b. As indicated in Figure 1c, the 3H-AE RNA tiles were modified with 120° KLs to enable formation of hexagonal assemblies from multiple interacting structures. Correct folding of the RNA tiles and their assembly into super-structures were assessed via atomic force microscopy (Figure 1d). In addition, 120° KL interactions between individual tiles resulted in the assembly of hexagonal lattices on the length scale of a few tens of nanometers. Larger, more disordered molecular networks were formed with hundreds of nanometers in size (Supplementary Figure S3). The dimensions of the RNA tiles observed via AFM were in accordance with the design of Figure 1a.

### 3.2. Modification of the 3H-AE RNA Tile with Aptamers

**Figure 2:**
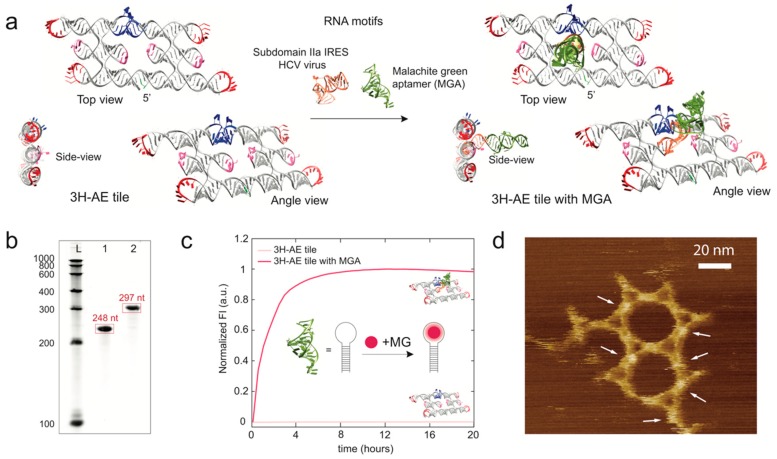
Modification of the 3H-AE RNA tile with the malachite green aptamer (MGA). (**a**) schematic representation showing different views of 3H-AE RNA tiles with and without the addition of MGA (green) connected via subdomain IIa in IRES of HCV virus (orange); (**b**) denaturing PAGE gel showing the length of the unmodified (no aptamer) and modified RNA tiles (with MGA). L: LowRange RiboRuler, 1: 3H-AE RNA tile, 2: 3H-AE RNA tile with MGA; (**c**) real-time fluorescence of MG recorded during transcription of MGA modified RNA tiles (fluorescence normalized to maximum value). The transcription reaction typically ceases after 3–4 h due to activity loss of the RNAP; (**d**) AFM image showing formation of a hexagonal mini-lattice with elevated features resulting from the MGA modifications (note that not all of the modifications are visible equally well, probably depending on the orientation of the extensions with respect to the AFM scanning direction). The sample was prepared by snap-cooling and incubation on mica as described in the Materials and Methods.

We next designed modified versions of the 3H-AE RNA tile with one or more fluorescent RNA aptamers. The first version included the replacement of one of the two interior tetraloops (sitting on one of the central RNA helices of the three-helix structure, (#2a) by two additional RNA motifs—subdomain IIa of the internal ribosomal entry site (IRES) of the HCV genome (PDB ID: 2PN4) and the malachite green aptamer (MGA) (Figure 2a). Subdomain IIa is an L-shaped structural motif that provides a 90° bend of the double helix center axis before and after the motif. The length of helix #2a and subdomain IIa together facilitated the attachment of an additional RNA domain protruding perpendicularly from the plane defined by the 3H-AE tile. As an example, for this attachment strategy, we elongated subdomain IIa with the fluorogenic MG aptamer (3H-AE with MGA V1, cf. Figure 2a).

This modification resulted in an increase in the number of nucleotides in the tile from 248 to 297 nt, which was verified by denaturing PAGE (Figure 2b). The presence of the fluorescent aptamer on the RNA tile further enabled real-time monitoring of the production of RNA tiles via in vitro transcription (Figure 2c). The stability of the signal over more than 20 h suggests negligible degradation of the RNA structure under our reaction conditions. AFM observation of small hexagonal assemblies by the MGA-functionalized tiles showed their correct folding and also demonstrated that the 120° KL interactions remained intact despite the modifications (Figure 2d).

We also created a variation of 3H-AE-MGA V1 by replacing the connecting motif subdomain IIa (PDB ID: 2PN4) by the slightly larger HCV IRES domain IIa (PDB ID: 1P5M), which includes a few additional unpaired bases. As a result, the length of the tile was increased by six bases from 297 nt to 303 nt (3H-AE-MGA V2), and the angle of MGA with respect to the tile was expected to tilt slightly (Supplementary Figure S4). Characterization by fluorescence spectroscopy and AFM showed very similar behaviors for both connection motifs (Supplementary Figure S4a,d).

### 3.3. Double Functionalization of the 3H-AE RNA Tile with Two Aptamers

**Figure 3:**
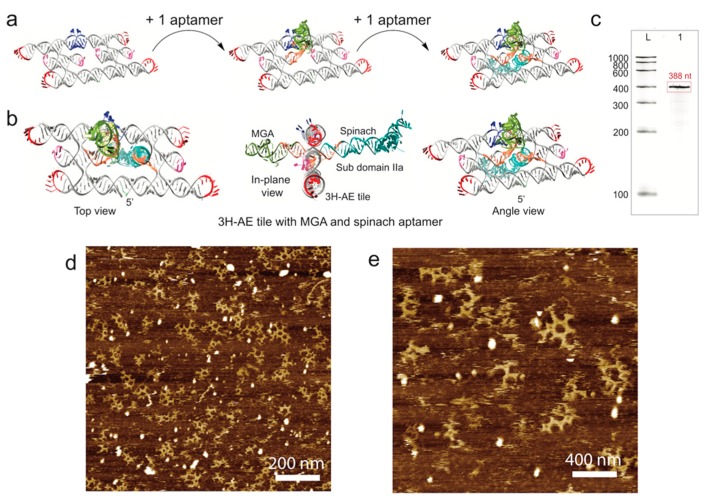
Modification of the MGA functionalized 3H-AE tile with an additional Spinach aptamer (resulting in 3H-AE-MGA-Spinach). (**a**) schematic representation of the addition of MGA (green) and Spinach aptamer (cyan) to the 3H-AE RNA tile connected via subdomain IIa (orange); (**b**) different views of 3H-AE-MGA-Spinach; (**c**) denaturing PAGE gel showing the correct length of 3H-AE-MGA-Spinach. L: LowRange RiboRuler, 1: 3H-AE-MGA-Spinach (388 nt); (**d**,**e**) AFM images corresponding to different areas of imaging—1 µm × 1 µm and 0.5 µm × 0.5 µm, respectively. Samples prepared by snap-cooling followed by incubation on mica.

Next, the complexity of the MGA-functionalized 3H-AE tiles was further increased by the addition of another aptamer via an additional subdomain IIa connected to the second internal tetraloop of the tile structure. As shown in Figure 3a,b, we used this approach to attach the fluorogenic Spinach aptamer (PDB ID: 4KZD) pointing perpendicularly away from the tile in the direction opposite to the initial MGA functionalization. Accordingly, the length of the tile’s RNA sequence increased to 388 nt, which was confirmed by denaturing PAGE (Figure 3c). AFM of the double-modified RNA tiles showed structures with local, 120° KL-mediated hexagonal order (Figure 3d,e). As expected, larger assemblies were not observed, as the extension of the aptamers in opposite directions did not allow the RNA tiles to lie flat on the mica surface. AFM imaging of the double-modified tiles therefore also turned out to be particularly challenging, in part probably due to the reduced contact area of the structures with the mica surface.

**Figure 4:**
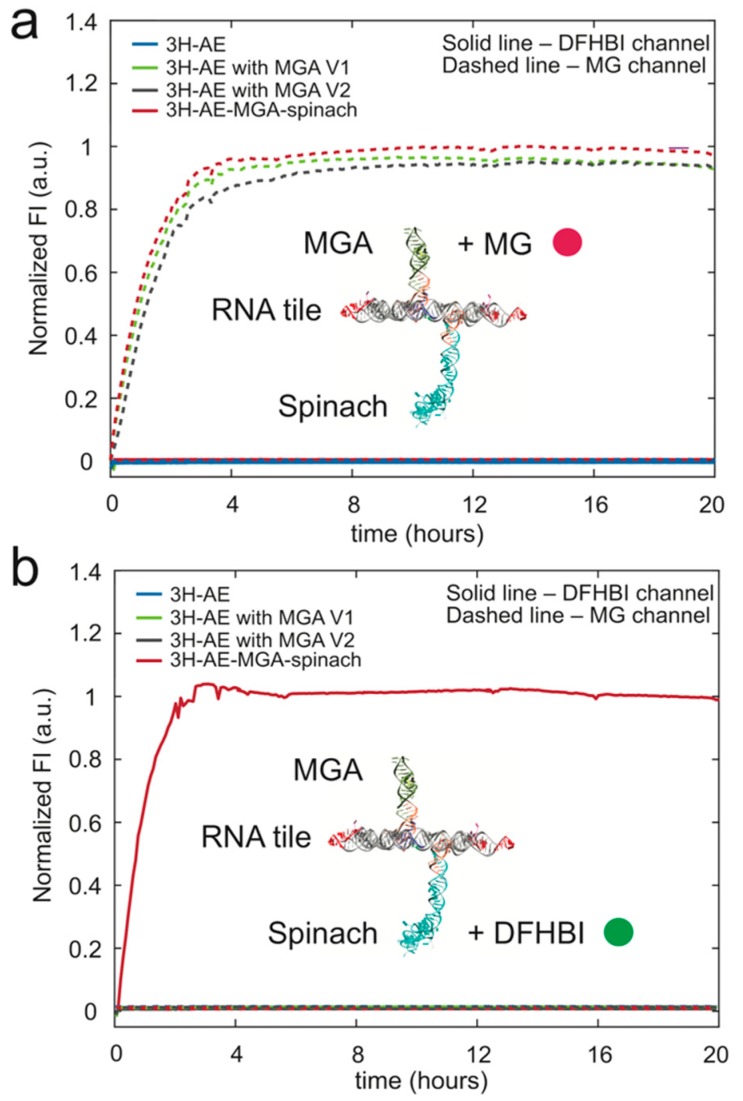
Monitoring transcription of RNA tiles via aptamer fluorescence. MG and the Spinach aptamer fluorophore 3,5-difluoro-4-hydroxybenzylidene imidazolinone (DFHBI) were added to transcription reactions of all four versions of RNA tiles. The structure of the 3H-AE-MG-Spinach-KL RNA tile is shown as an inset to the figures: (**a**) MGA and (**b**) Spinach (DFHBI) fluorescence recorded during transcription of the various RNA tiles constructed (Ex = 615–645 nm, Em = 669–699 nm for MG and Ex = 475–495 nm, Em = 515–545 nm for DFHBI; fluorescence intensities normalized with respect to maximum fluorescence).

As before, transcription of the double-modified RNA tile was monitored via the fluorescence signal generated by the two aptamers in their respective emission channels (Figure 4), indicating proper folding of the aptamer domains and stability of the structure.

We also designed a modification of 3H-AE-MGA-Spinach-KL, in which the 120° KLs were replaced by tetraloops to avoid inter-tile interactions, keeping all the other features unaltered. The non-interacting tile displayed similar behavior in native gel electrophoresis and fluorescence experiments (Supplementary Figures S5–S7). An enhanced fluorescence value observed for the tetraloop-containing structure (Supplementary Figure S6c) potentially indicates a slightly better folding of the tile, which would be consistent with the fact that tetraloops support the intramolecular folding process. Alternatively, it is possible that the two structures are transcribed at different rates given the different sizes and base compositions of the sequences.

**Figure 5:**
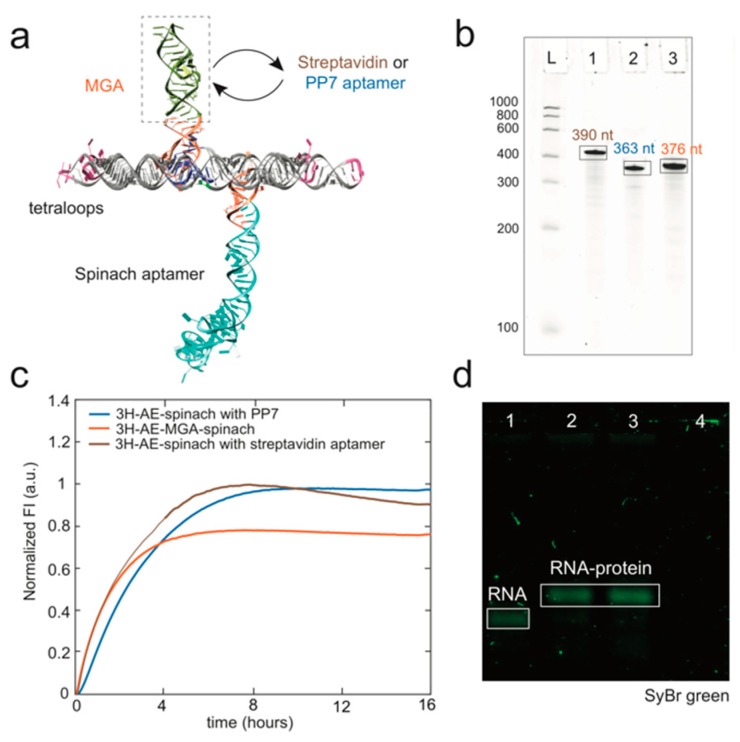
(**a**) schematic representation of 3H-AE-MGA-Spinach RNA tile without 120° KL. The MG aptamer can be modularly replaced by other RNA aptamers as indicated; (**b**) denaturing PAGE gel showing the length of 3H-AE-Spinach with different secondary aptamer modifications. L: LowRange RiboRuler, 1: 3H-AE-Spinach with streptavidin aptamer (390 nt), 2: 3H-AE-Spinach with PP7 aptamer (363 nt), 3: 3H-AE-MGA-Spinach (376 nt); (**c**) real-time fluorescence of DFHBI during transcription of RNA tiles containing different aptamers; (**d**) native agarose gel (2% agarose in 1X tris-borate EDTA (TBE) buffer + 2 mM MgCl_2_) stained with SyBr Green showing the retardation of RNA tile containing streptavidin aptamer (3H-AE-Spinach with streptavidin aptamer) in presence of streptavidin. 1: RNA tile only, 2: 3H-AE-Spinach with streptavidin aptamer + streptavidin (100 nM), 3: 3H-AE-Spinach with streptavidin aptamer + streptavidin (200 nM), 4: Streptavidin only (100 nM). RNA tiles were prepared by heat denaturation/renaturation as described in the Supporting Materials and Methods.

For specific applications, the fluorogenic aptamer modifications can be easily replaced with other functional RNA sequences. As a proof of concept, two variants of 3H-AE-MGA-spinach-Tetraloop structure were designed and constructed with the MGA replaced by the PP7 aptamer [28] or a streptavidin aptamer, respectively (Figure 5a and Figure S8). The production of the structures was validated by real-time fluorescence of the (remaining) spinach aptamer and denaturing PAGE (Figure 5b,c). Interactions of the tiles with streptavidin (Figure 5d) and PCP-mCherry fusion proteins (Supplementary Figures S9–S11) were further verified by EMSA.

**Figure 6:**
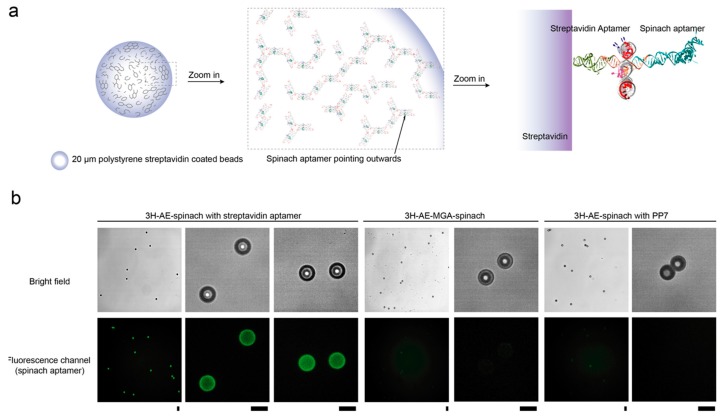
Assembly of double-functionalized 3H-AE tiles on microparticles. (**a**) schematic representation of attachment of 3H-AE-Spinach tiles with streptavidin aptamer on the surface of streptavidin coated beads. The streptavidin aptamer is expected to be anchored on the surface of streptavidin particles and thus the Spinach aptamer points away from it; (**b**) fluorescence images of different RNA tiles interacting with 20 µm streptavidin coated polystyrene beads after washing. Only the RNA tiles carrying Spinach and a streptavidin aptamer lead to a fluorescent signal localized to the beads; scale bars: 20 µm.

In order to indicate one potential application for double-functionalized 3H-AE tiles, we utilized the streptavidin aptamer module to specifically arrange RNA tiles on the surface of streptavidin-coated polystyrene microbeads. 3H-AE tiles double-functionalized with the Spinach and streptavidin aptamer are expected to present the Spinach aptamer in an orientation pointing away from the surface of the beads (Figure 6a). Indeed, binding of the RNA tiles to the microparticles is observed only with nanostructures containing the streptavidin aptamer (Figure 6b). While in this experiment the fluorogenic Spinach aptamer was used as the second function on the RNA tile, it is easily conceivable to generate protein-functionalized surfaces or membranes using other aptamer modules in a similar manner. Such surfaces should be of interest for the spatial organization of enzymes or other biochemical functions. As the formation of such functionalized surfaces could be triggered by the transcription of the RNA tiles, this process would also be genetically controllable.

## 4. Conclusions

The results demonstrated here represent initial efforts towards the design and synthesis of multifunctional tile-based RNA nanostructures extending in three dimensions. Three-helix RNA tiles were folded from a single RNA strand, which was composed of a variety of naturally occurring RNA motifs that assist in reaching the desired target shape. The main focus of the present work was put on domain IIa and subdomain IIa, which are RNA motifs found in the HCV IRES. Both these motifs contain a 90° bend that allows positioning of other RNA modules such as fluorogenic RNA aptamers perpendicular to the RNA tile structure. As examples, we created 3H-AE tile structures either modified with a single malachite green aptamer or with MGA and a Spinach aptamer pointing in opposite directions. Importantly, the modified 3H-AE-tiles retained the ability to associate into hexagonal assemblies via kissing loop interactions.

Among the most promising applications of such multi-functionalized RNA tile structures, we envision the creation of artificial ribonucleoprotein complexes, in which RNA structures scaffold the co-localization and arrangement of proteins, e.g., for membrane-less compartmentalization or for the creation of multienzyme structures. As the RNA tiles can be made to polymerize in 2D, it is also conceivable to create extended RNA sheets or “membranes”, which are functionalized with aptamers on either side, which could be used to further spatially organize proteins in order to enhance their structural or enzymatic functions. From these, in turn, one could generate RNA/protein covered surfaces (e.g., with catalytic function) via self-assembly of such structures. Importantly, such self-assembly processes could all be controlled via gene expression from a plasmid. From a nanotechnology point of view, another interesting opportunity lies in the possibility to stack several such RNA sheets on top of each other—mediated by the perpendicular RNA extensions, resulting in multilayered RNA nanostructures extending in 3D.

## Supplementary Materials

The following are available online at https://www.mdpi.com/2079-4991/9/4/507/s1, Supplementary Methods and Materials, Supplementary Figures S1–S11, Supplementary Tables S1–S7, Supplementary References 1–3.
