# Supplementary material for: Out-of-Plane Aptamer Functionalization of RNA Three-Helix Tiles
Source: Nanomaterials (Basel). 2019 Apr 2;9(4):507. doi: 10.3390/nano9040507 (PMC6523889; doi:10.3390/nano9040507)
Supplement: Supplementary file 1 [file nanomaterials-09-00507-s001.pdf]

Supplementary Materials

Out-of-Plane Aptamer Functionalization of RNA Three-helix Tiles

Aradhana Chopra [1], Sandra Sagredo [1], Guido Grossi [2], Ebbe S. Andersen [2] and Friedrich C. Simmel [1,*]

[1] Physik-Department E14, Technische Universität München, 85,748 Garching, Germany
[2] Interdisciplinary Nanoscience Center (iNANO), Aarhus University, 8000 Aarhus C, Denmark
[*] Correspondence: simmel@tum.de; Tel: +49-(0)89-289-11611; Fax: +49-(0)89-289-11612

1. Supplementary Methods & Materials

Polymerase chain reaction (PCR) – DNA templates for all RNA designs were ordered as "custom dsDNA gBlocks" from Integrated DNA Technologies (IDT). Custom gBlocks were supplied as 250 ng of lyophilized DNA. DNA gBlocks were reconstituted in nuclease free water (nF water) to a concentration of 5 ng/µL and were amplified by Phusion PCR master mix (2X) from New England Biolab (NEB) over 25 cycles using primers specific to the 5′ and 3′ ends of the template in a Mastercycler (Eppendorf). Amplified DNAs were purified using a standard Monarch PCR Cleanup kit from NEB and suspended in nF water. PCR products were verified using 2% agarose gel containing SERVA DNA stain G (Serva). The gel was run in 1X TAE at room temperature at 120 V for 45 min. Gel images were taken on a Typhoon FLV 9000 laser scanner and analyzed with the software package Fiji [1]. DNA gBlocks, primer sequences and RNA sequences for all the tile designs along with the corresponding conditions used for PCR amplification are listed in Tables S1–S4 provided in the supplementary information respectively.

In vitro transcription – RNA tiles for different experiments were transcribed in vitro using T7 RNA polymerase (T7RNAP) and purified. Approx. 5 ng of template DNA was mixed in a transcription reaction containing 1X RNA polymerase buffer (RNAP buffer) and 16 mM rNTPs (4 mM each) from NEB. 15 mM $MgCl_2$ and RNase Inhibitor (NEB) (≈1 U/µL) were also added prior to addition of 200 nM T7 RNAP. T7 RNAP was expressed and purified in-house by S. Sagredo and had a concentration of 10 µM. Alternatively, T7 RNAP can be used from a commercial supplier. Reactions were carried out in 50 µL volumes at 37 °C for 3–4 h. For RNA designs with aptamer modifications, non-fluorescent chemical compounds Malachite green (MG) (10 µM) and DFHBI (3,5-difluoro-4-hydroxybenzylidene imidazolinone, (10 µM) respectively were also added that emitted fluorescence only in presence of the respective MG and spinach aptamers. Additionally, 125 mM potassium chloride (KCl) was added during transcription of the RNA tile with spinach aptamer modification. Transcription of RNA tiles with modifications was monitored with time by measuring the fluorescence of MG and DFHBI using iQ5 real-time PCR detection system from Bio-Rad using filters with excitation and emission wavelengths corresponding to the fluorophores (Ex = 475–495 nm, Em = 515–545 nm for DFHBI and Ex = 615–645 nm, Em = 669–699 nm for MG) [2].

Purification of the structures – Prior to purification, the transcription reaction was stopped by addition of 0.08 U/µL DNase I (NEB) and incubated for 30 min at 37 °C. Purification was performed by standard phenol-chloroform extraction using Phase Lock Gel (PLG) heavy columns from QuantaBio. RNAs were precipitated by 2–3 volumes 100% ethanol (EtOH) and 0.1 volume 5 M sodium acetate (pH 5.5) for at least one hour followed by subsequent washing in 70% EtOH, drying under vacuum and resuspension 20 µL nF water. An additional purification step was carried out to separate the desired product from abortive transcripts using Quantum Prep Freeze 'N Squeeze gel extraction spin columns from Bio-Rad. RNA samples were run on denaturing polyacrylamide gel electrophoresis (PAGE) and the correct bands corresponding to the RNA tiles were cut and put in Quantum Prep Freeze 'N Squeeze gel extraction spin columns for 15 min at −20 °C and centrifuged for three times at 13,000 rcf for 6 min at room temperature. Further, the RNA samples were

concentrated using Eppendorf Concentrator 530f and the concentration was estimated by denaturing polyacrylamide gel electrophoresis (denaturing PAGE).

Polyacrylamide gel electrophoresis (PAGE) – 8% Acrylamide: Bis (29:1) gel containing 8 M urea and 1X TBE buffer (denaturing PAGE) was used to analyze the purified RNA tiles and estimate their concentration. The gel was pre-run at 50 °C for 30 min. The samples and RiboRuler Low Range Ladder (Thermofisher scientific) were prepared in 1:1 ratio with 2X RNA loading dye (Thermofisher scientific) and incubated at 80 °C for 10 min prior to loading onto the gel. The gels were run at 80 V for 1 h 45 min. After electrophoresis, the gels were stained with SYBR™ Green II RNA Gel Stain (Thermofisher scientific) for 10 min and scanned on a Typhoon FLV 9000 laser scanner and analyzed with the software package Fiji [1].

Native PAGE – 8% Acrylamide: Bis (29:1) gel containing 1X TBE buffer and 2 mM $(MgOAc)_2$ was used to analyze the proper folding of purified folded RNA tiles. The gel was pre-run at 4 °C for 30 min. The tiles were subjected to a heat denaturation/renaturation protocol (95° for 3 min and then snap-cooled to 4 °C for 5 min) and buffer was added (1X buffer = Tris-Borate 1X (pH 8.1), 2 mM magnesium acetate ($Mg(OAc)_2$), 50mM potassium chloride (KCl), 50 mM sodium chloride (NaCl)). The samples were either kept on ice or further incubated for 1 h at 37 °C prior to loading onto the gel. The gel was run at 80 V for 1 h 30 min at 4 °C. After electrophoresis, the gels were stained with 10 µM Malachite green for 30 min and scanned on a Typhoon FLV 9000 laser scanner. Additionally, the gel was stained with SYBR™ Green II RNA Gel Stain (Thermofisher scientific) for 10 min and scanned on a Typhoon FLV 9000 laser scanner. The gel was analyzed with the software package Fiji [1].

1.1. EMSA

1.1.1. 3H-AE-Spinach with Streptavidin Aptamer

2% agarose gel in 1X TBE and 2 mM $MgCl_2$ were used to determine the shift in RNA tile migration after being bound to streptavidin via its RNA aptamer. The gel was run in ice water bath at 80 V for 2 h. 100 nM RNA tiles were subjected to a heat denaturation/renaturation protocol as mentioned previously and then incubated with different concentrations of streptavidin (NEB, 1mg/mL, MW: 52.8KDa, ~19 µM) for 10–15 min in presence of 1X AFM buffer and Murine RNase Inhibitor (NEB) at room temperature. 40% sucrose was used for loading the samples onto the gel. After EMSA, the gel was stained with SYBR™ Green II RNA Gel Stain (Thermofisher scientific) for 30 min and destained. It was scanned on a Typhoon FLV 9000 laser scanner and analyzed with the software package Fiji [1].

1.1.2. 3H-AE-Spinach with PP7 RNA Aptamer

2% agarose gel in 1X Tris-Glycine buffer (25 mM Tris, 192 mM Glycine, 10 mM KCl, 2 mM $MgCl_2$, pH 8.1) was used to determine the shift in RNA tile migration after being bound to PCP-mCherry via its PP7 RNA aptamer. The gel was run in ice-water bath at 200 V for 35 min. The gel was pre-run at 10 V for 30 min. 100 nM RNA tiles were subjected to a heat denaturation/renaturation protocol as mentioned previously and then incubated with different concentrations of PCP-mCherry (~4 µM) (details about expression and purification are provided in the next section) for 10–15 min in presence of 1X AFM buffer and Murine RNase Inhibitor (NEB) at RT. 40% sucrose was used for loading the samples onto the gel. After EMSA, the gel was scanned first at wavelength corresponding to mCherry fluorescence, stained with SYBR™ Green II RNA Gel Stain (Thermofisher scientific) for 30 min and destained. It was scanned on a Typhoon FLV 9000 laser scanner and analyzed with the software package Fiji [1].

1.2. Gibson Assembly and Protein Expression

A DNA template encoding the PCP coat protein (insert) was ordered as gBlocks from IDT and cloned into the pET28 backbone containing mCherry fluorescent protein by Gibson Assembly (NEB E2611S) following the manufacturer’s protocol. Primer sequences along with the corresponding conditions used for PCR used for Gibson Assembly are listed in Tables S5 and S6 in the

supplementary information. The plasmid maps and other relevant sequences are provided in Figure S10 and Table S7. The product of Gibson assembly was electroporated in electro competent Dh5α *E. coli* cells. Plasmid was isolated from an overnight culture of Dh5α containing the pET28-PCP-mCherry plasmid and sequenced for the correctness of the insert. Further, purified pET28-PCP-mCherry plasmid was electroporated in BL21DE3star *E. coli* for protein expression.

100 mL LB medium was inoculated with an overnight culture of BL21DE3star *E. coli* containing pET28-PCP-mCherry plasmid and was induced with 1 mM IPTG at OD = 0.6. The culture was allowed to grow for 3–4 h and pelleted. The pellet was resuspended in lysis buffer (50 mM Tris-NaOH, 500 mM NaCl, 5 mM imidazole, 1 mM Benzamidine and 0.1% PMSF) and sonicated (Amplitude: 50%, Pulse: 30 s, time: 10 min) on ice. It was further centrifuged and the supernatant was collected and loaded onto Ni-NTA column for purification by AKTA as PCP-mCherry protein included a His-tag (6xHis). The protein was eluted at ~400 mM imidazole concentration. The fractions collected from AKTA were run on a SDS-PAGE gel. 2X Laemmli buffer was added to the fractions and they were incubated at 95 °C for 3 min. 12% SDS-PAGE gel was run at 250 V for 25 min in 1X SDS buffer as running buffer. The protein was washed in equilibration buffer (50 mM Tris-NaOH, 500 mM NaCl, 1 mM EDTA) 3–4 times at 4000 rcf for 10 min at 4 °C using 10 kD cut off filters from EMD millipore. 10% glycerol was added to make the stocks and kept at −80 °C. The concentration of the protein was determined by the absorbance measured by UV-Vis spectrophotometer and the protein parameters determined by Expasy [3].

1.3. Sample Preparation and Atomic Force Microscopy (AFM)

Freshly-cleaved mica affixed to a metal disk was placed inside an empty tip box containing a wet Kimwipe and water (to maintain the humidity in the chamber). A 60 μL drop of 1.25X AFM buffer (1X AFM buffer = Tris-Borate 1X (pH 8.1), 2 mM magnesium acetate ($Mg(OAc)_2$), 50 mM potassium chloride (KCl), 50 mM sodium chloride (NaCl)) was added to the mica and incubated at 37 °C. Purified RNA tiles (after Freeze and Squeeze gel extraction) were diluted in pure water at a concentration calibrated to give coverage, but not large aggregates, on the mica surface (~75–100 nM for 3H tiles). Tiles were next subjected to a heat denaturation/renaturation protocol (95° for 3 min and then snap-cooled to 4 °C for 5 min). 15 μL of RNA sample was added to the mica on top of the pre-heated (37 °C) buffer drop and incubated for 45 min to 1 h to allow coverage of the surface and formation of hexagonal lattices. AFM images were collected in tapping mode under buffer using Asylum research's high resolution fast scanning AFM Cypher S. Olympus BL-AC40TS-C2 silicon nitride probes with a spring constant of ~0.09 N/m and at a resonant frequency of ~20–30 kHz was typically used for imaging, with a drive frequency of ~5–8 kHz.

1.4. Inmobilization of RNA Tiles on Streptavidin-coated Beads and Imaging

For studying the arrangement of RNA tiles on the surface of microbeads, we used 20 μm streptavidin polystyrene coated beads (Spherotech). Beads were washed three times for 10 min with nuclease free water and 5 μL were transferred to 30 μL transcription reactions and incubated for 1 h at 37 °C. Transcription reactions were performed as previously described for 6–8 h. Samples were washed once with 1x TX reaction to remove the unspecific binding and resuspended in 20 μL transcription buffer with 100 μM DFHBI (40 mM Tris-HCl pH 7.9, 100 mM $MgCl_2$, 125 nM KCl, 1 mM DTT, 2 mM spermidine). Samples were imaged with a Nikon Ti-Eclipse epi-fluorescence microscope controlled with NIS-Elements Imaging Software. The microscope was equipped with a SOLA SM II LED light source, a sCMOS camera (Zyla, Andor), a motorized stage (Prior Scientific, Cambridge, UK), and a perfect focus system

2. Supplementary Figures

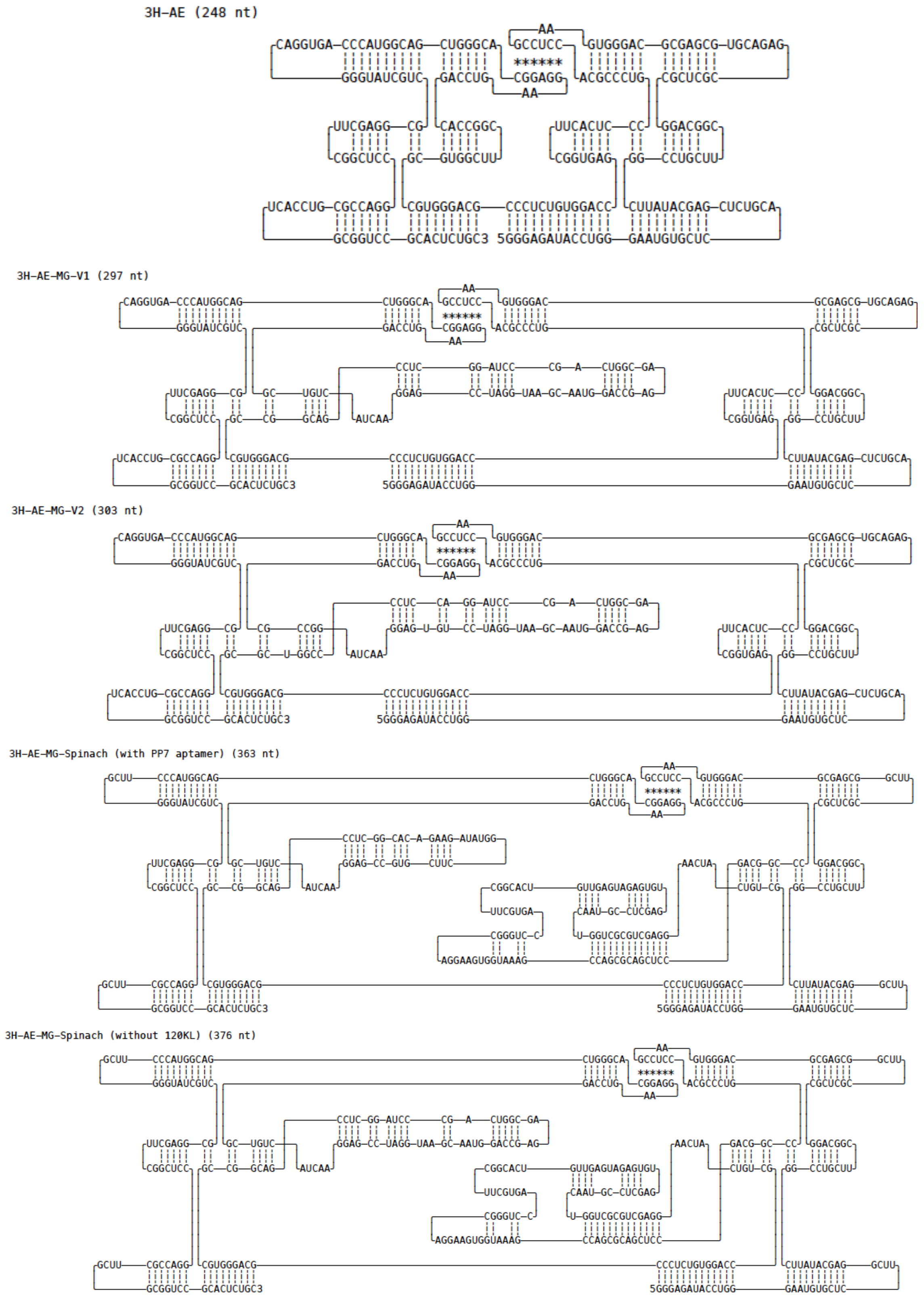

Figure S1. *Cont.*

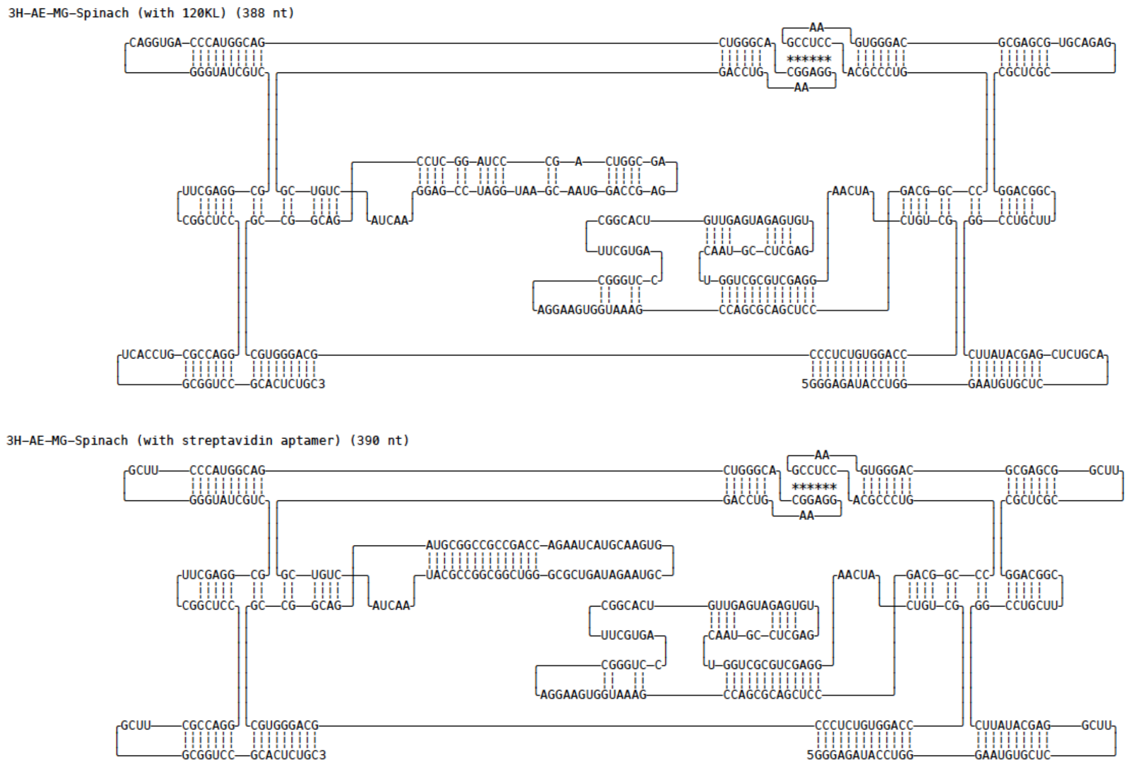

Figure S1. 2D blueprints of the RNA tiles reported in this work.

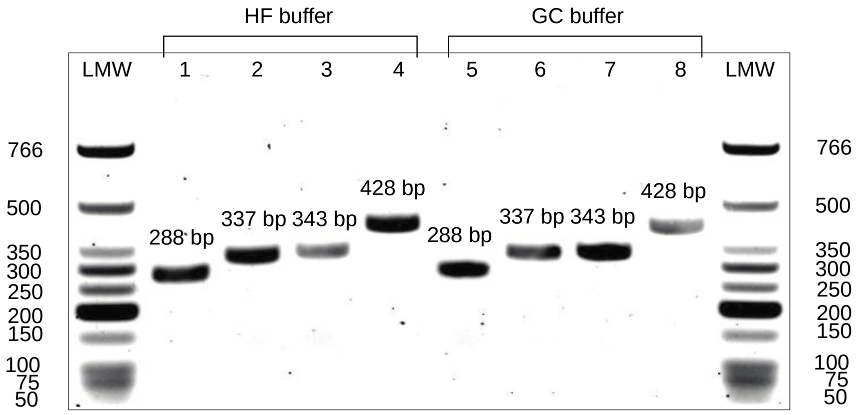

Figure S2. Verification of PCR products corresponding to DNA templates for different RNA tiles. 2% agarose gels show the correctness of length and the purity of the PCR products. LMW: Low Molecular Weight Ladder, 1-4: amplified DNA template (in HF Phusion master mix) 3H-AE (288 bp), 3H-AE with MGA V1 (337 bp) and V2 (343 bp) and 3H-AE with MGA and spinach aptamer (388 bp) respectively, 5-8: amplified DNA template (in GC Phusion master mix) 3H-AE, 3H-AE with MGA V1 and V2 and 3H-AE with MGA and spinach aptamer respectively.

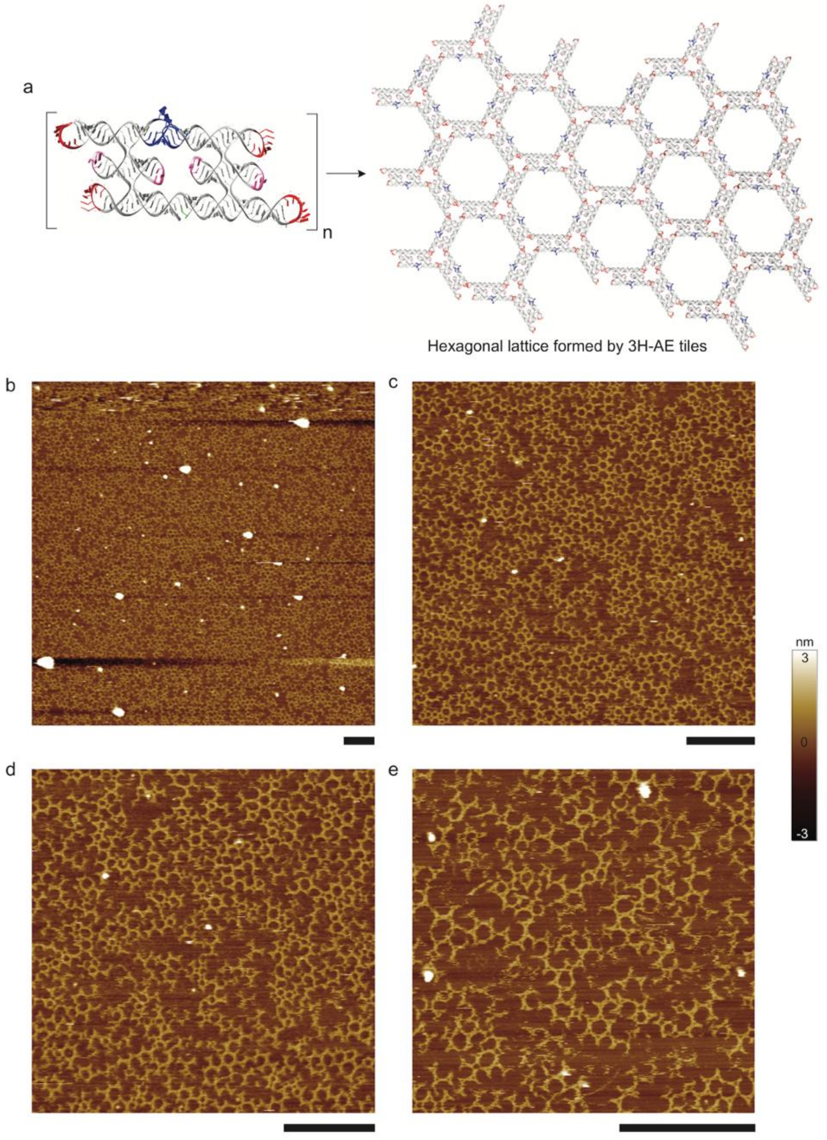

Figure S3. Formation of hexagonal assemblies by 3H-AE RNA tiles via 120° KL interactions. (**a**) Schematic representation of the formation of hexagonal assemblies. (**b**–**e**) AFM images showing hexagonal assemblies and small lattices, corresponding to different areas of imaging—2 μm × 2 μm, 1 μm × 1 μm, 700 nm × 700 nm and 500 nm × 500 nm respectively. Scale bar: 200 nm. Samples were prepared by snap-cooling followed by incubation on mica as described in the SI Materials & Methods.

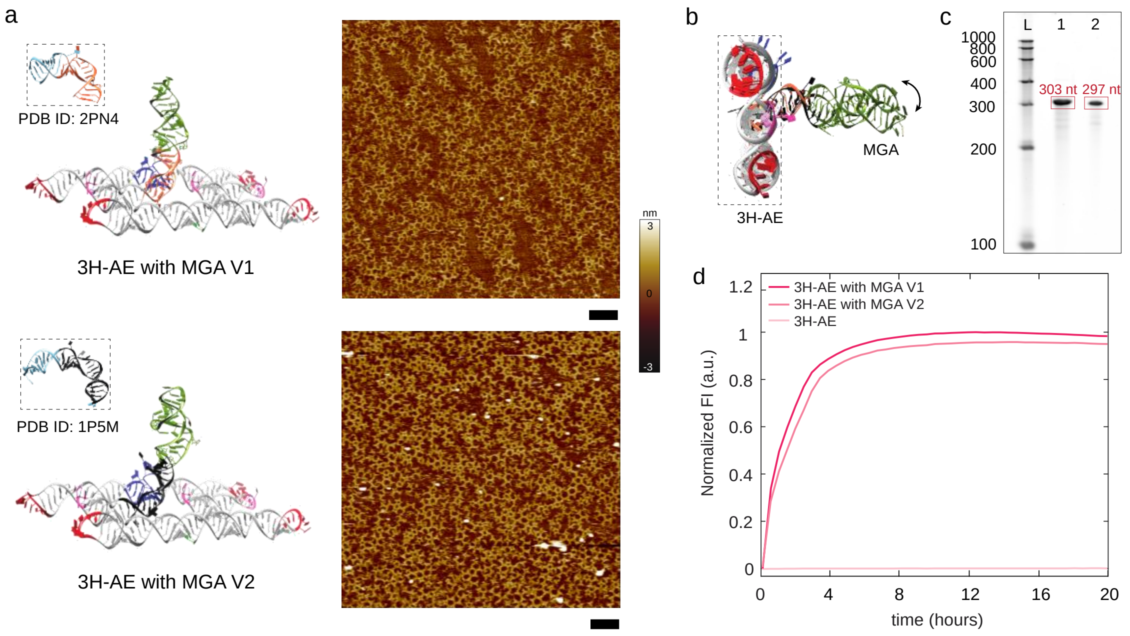

Figure S4. Comparison of 3H-AE with MGA V1 and V2. (**a**) Schematic and corresponding AFM images of 3H-AE with MGA V1 and V2, respectively. Samples prepared by snap cooling as described in the SI Materials & Methods. Area of imaging – 1 µm × 1 µm. MGA is connected to the tiles via subdomain IIa (PDB ID: 2PN4, coral region) (also seen in Figure 2) and domain IIa (PDB ID: 1P5M, black region) of IRES in HCV virus respectively. Inset shows the extracted motif (sky blue). (**b**) Side-view of the overlay of V1 and V2, showing a slight shift in the angle between MGA and RNA tile (arrow) due to the presence of two additional base pairs and two unpaired bases. (**c**) 8% Denaturing PAGE gel showing 3H-AE with MGA. L: LowRange RiboRuler, 1: 3H-AE with MGA V2 (303 nt), 2: 3H-AE with MGA V2 (297 nt). (**d**) Real-time fluorescence measurements demonstrating co-transcriptional folding of the RNA tile with MGA. Scale bar: 50 nm.

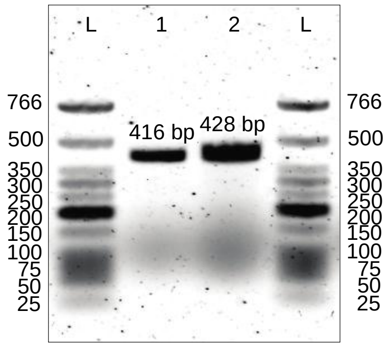

Figure S5. Verification of PCR products corresponding to DNA templates for 3H-AE-MGA-spinach with and without 120° KL. 2% agarose gels showing the correctness of length and purity of the PCR products amplified using HF Phusion master mix. LMW: Low Molecular Weight Ladder, 1: 3H-AE-MG-spinach without 120° KL (416 bp), 2: 3H-AE-MG-spinach without 120° KL (428bp).

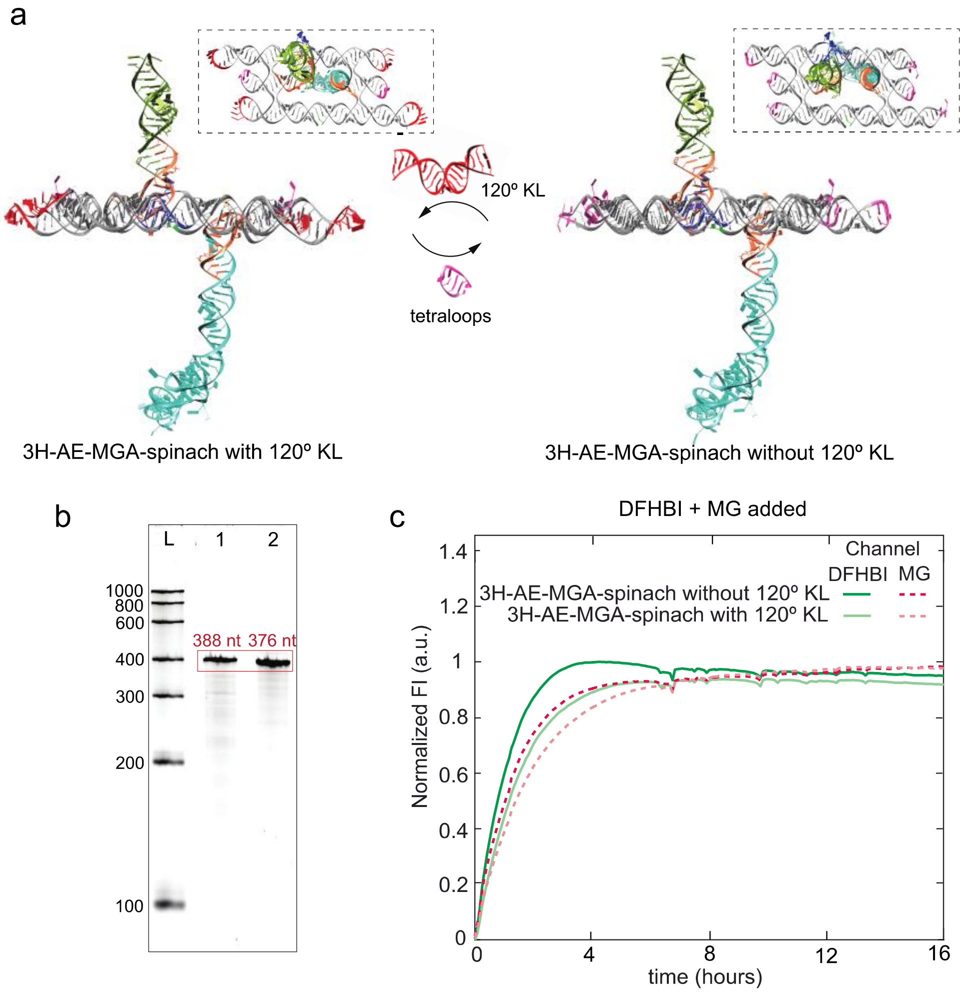

Figure S6. (**a**) Schematic representation of the 3H-AE-MGA-spinach RNA tile with the 120° KLs (red) replaced by tetraloops (pink). (**b**) Denaturing PAGE gel showing the length of 3H-AE-MGA-spinach with and without 120° KL. L: LowRange RiboRuler, 1: 3H-AE-MGA-spinach with 120°KL (388 nt), 2: 3H-AE-MGA-spinach without 120° KL (376 nt). (**c**) Real-time fluorescence of MG and DFHBI fluorescence during transcription of the 3H-AE-MGA-spinach RNA tiles.

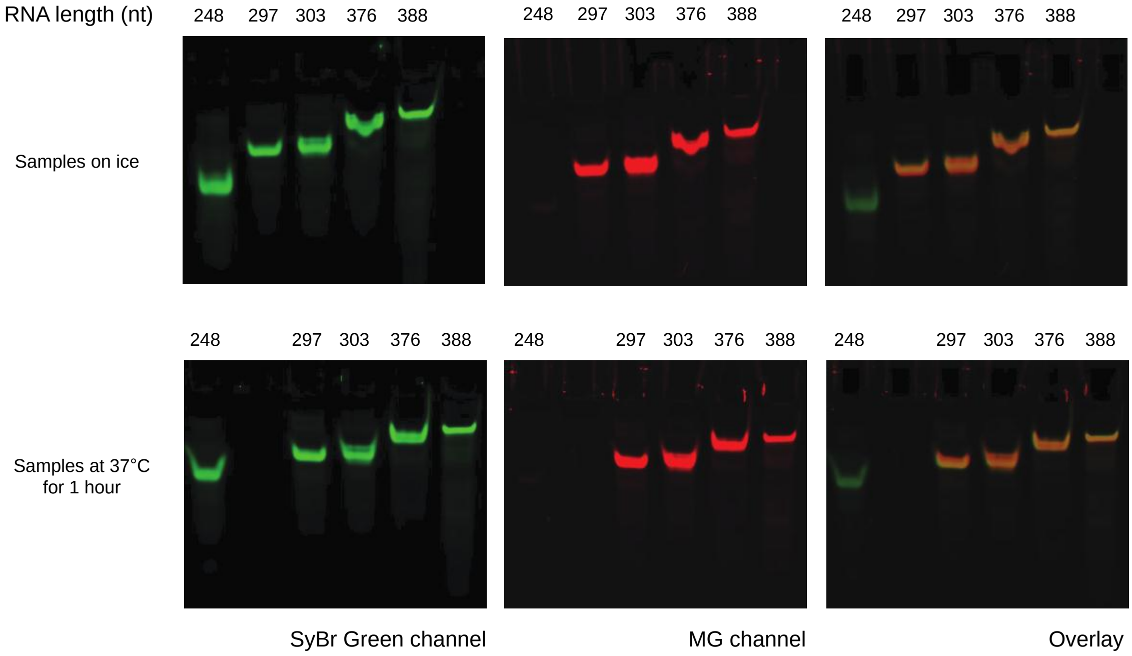

Figure S7. Native PAGE gels of RNA tiles observed in a fluorescence laser scanner: 248 nt: unmodified 3H-AE, 297 nt: 3H-AE-MG V1, 303 nt: 3H-AE-MG V2, 376 nt: 3H-AE-MG-Spinach-Tetraloop, 388 nt: 3H-AE-MG-Spinach-KL. After annealing with a temperature ramp, the purified structures were either kept on ice (top row) or further incubated at 37 °C for 1 h and then loaded onto the gel (bottom row). The gel was run at 4 °C for 1 h 30 min. The gel was stained with 10 μM MG for 30 min and scanned at MG fluorescence wavelength. Following that, the gel was stained with SyBr green and scanned. Except for the unmodified (248 nt) structure, all MGA-modified aptamers displayed fluorescence in both channels.

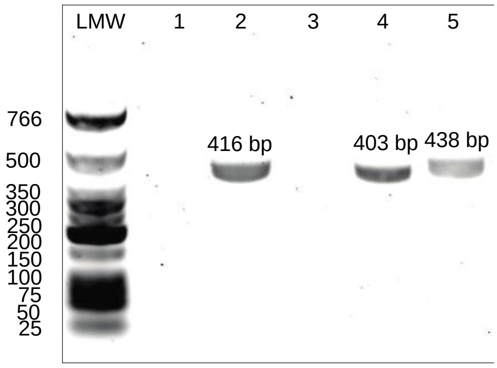

Figure S8. Verification of PCR products corresponding to DNA templates for different RNA tiles. 2% agarose gels showing the correctness of length and purity of the PCR products amplified using HF Phusion master mix. LMW: Low Molecular Weight Ladder, 2: 3H-AE-MG-spinach without 120° KL (416 bp), 4: 3H-AE-PP7-spinach without 120° KL, 5: 3H-AE-streptavidin-spinach without 120° KL (438 bp).

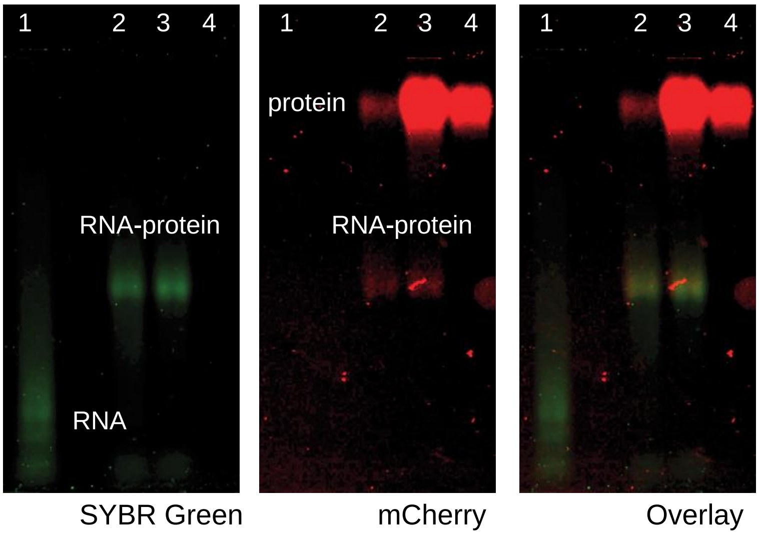

Figure S9. Electromobility Shift Assay (EMSA) showing the retardation of 3H-AE-spinach-PP7 RNA tile (without 120° KL) in the presence of PCP-mCherry fusion protein. 1: 3H-AE-spinach-PP7 RNA tile only (363 nt, c = 0.1 μM), 2: 3H-AE-spinach-PP7 RNA tile (0.1 μM) + PCP-mCherry (0.2 μM), 3: 3H-AE-spinach-PP7 RNA tile (0.1 μM) + PCP-mCherry (0.4 μM), 4: PCP-mCherry (0.2 μM) only. The gel was first scanned in mCherry fluorescence and following that it was stained with SyBr green and scanned again.

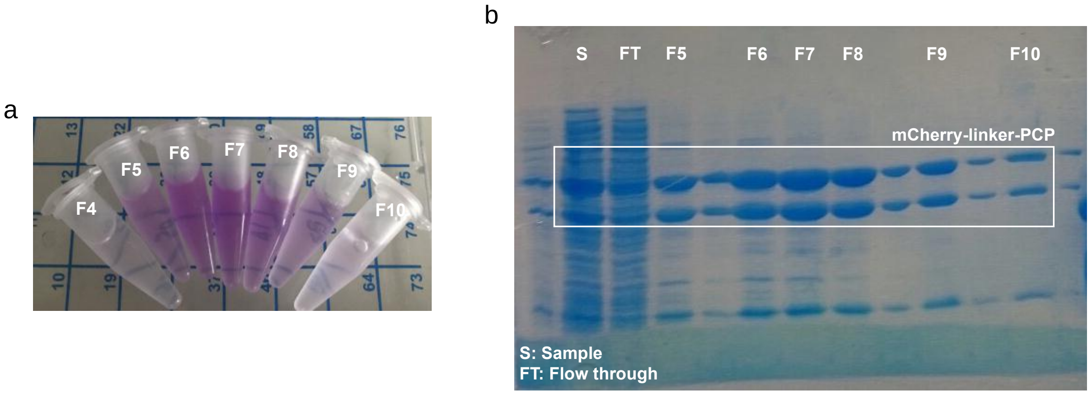

Figure S10. (**a**) Fractions containing mCherry-linker-PCP protein collected from ÄKTA. (**b**) SDS-PAGE gel showing mCherry-linker-PCP protein from different fractions.

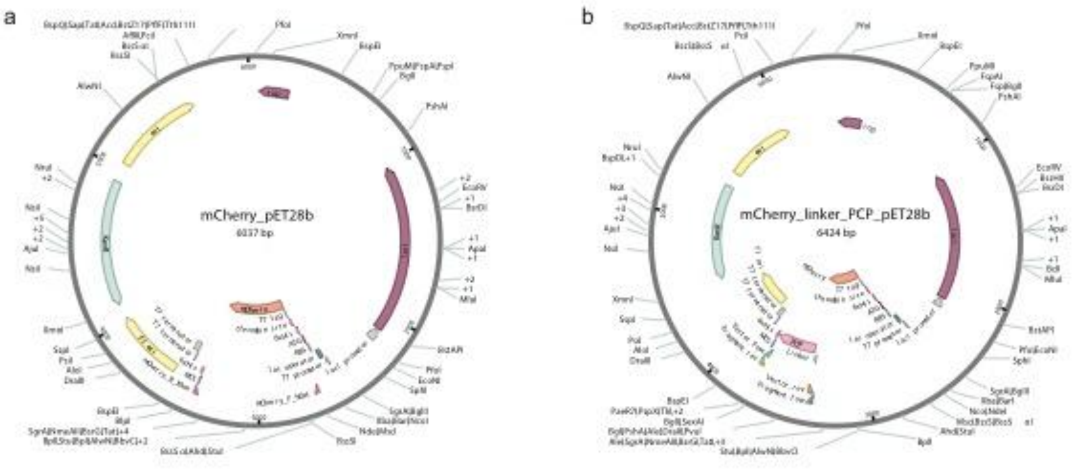

Figure S11. Plasmid maps. (**a**) mCherry-pET28b (vector) (6047 bp). (**b**) mCherry-linker-PCP-pET28b (vector + insert) (6424 bp). The linker-PCP (insert) was added after mCherry sequence by Gibson Assembly and was connected to it via a linker.

Table S1. DNA gBlock sequences and lengths corresponding to different RNA tiles. Red and green: Sequences complementary to the forward and reverse primer respectively. Blue: Promoter sequence from T7 RNA Polymerase. Black: Sequence encoding the RNA tile.

	Length (nt)	Sequence (5'-3')
3H-AE	288	AACTCTCGATCAGCGGACAGCGCTAATACGACTCACTATAGGGAGATACCTGGGAATGTGCTCACGTCTCGA<br>GCATATTCGGCCTGCTTCGGCAGGCGCTCGCGAGACGTGCGAGCGCAGGGTGAAGCCTCCACGCCCTGCC<br>CTCACTTCGGTGAGCCAGGTGTCTCCCGCAGGGTGCGCGTGGCTTCGGCCACGACCTGAAGGAGGCACGG<br>GTCGACGGTACCCAGTGGACGGGTATCGTCGCGGAGCTTCGGCTCCGGACCGCGTCCACTGCGGTCCGCAC<br>TCTGC
3H-AE-MG-V1	337	AACTCTCGATCAGCGGACAGCGCTAATACGACTCACTATAGGGAGATACCTGGGAATGTGCTCACGTCTCGA<br>GCATATTCGGCCTGCTTCGGCAGGCGCTCGCGAGACGTGCGAGCGCAGGGTGAAGCCTCCACGCCCTGCCC<br>TCACTTCGGTGAGCCAGGTGTCTCCCGCAGGGTGCGCCGGCAGCCTCGGATCCCGACTGGCGAGAGCCAGG<br>TAACGAATGGATCCGAGGAACTACTGTCGGACCTGAAGGAGGCACGGGTCGACGGTACCCAGTGGACGGGTA<br>TCGTCGCGGAGCTTCGGCTCCGGACCGCGTCCACTGCGGTCCGCACTCTGC
3H-AE-MG-V2	343	AACTCTCGATCAGCGGACAGCGCTAATACGACTCACTATAGGGAGATACCTGGGAATGTGCTCACGTCTCGAG<br>CATATTCGGCCTGCTTCGGCAGGCGCTCGCGAGACGTGCGAGCGCAGGGTGAAGCCTCCACGCCCTGCCCTC<br>ACTTCGGTGAGCCAGGTGTCTCCCGCAGGGTGCGCGCTGGCCCCTCCAGGATCCCGACTGGCGAGAGCCAGG<br>TAACGAATGGATCCTGTGAGGAACTAGGCCGCGACCTGAAGGAGGCACGGGTCGACGGTACCCAGTGGACGGG<br>TATCGTCGCGGAGCTTCGGCTCCGGACCGCGTCCACTGCGGTCCGCACTCTGC
3H-AE-MG-spinach (with 120°KL)	428	AACTCTCGATCAGCGGACAGCGCTAATACGACTCACTATAGGGAGATACCTGGGAATGTGCTCACGTCTCGA<br>GCATATTCGGCCTGCTTCGGCAGGCGCTCGCGAGACGTGCGAGCGCAGGGTGAAGCCTCCACGCCCTGCCCG<br>GCAGCCTCGACGCGACCGAAATGGTGAAGGACGGGTCCAGTGCTTCGGCACTGTTGAGTAGAGTGTGAGCTCC<br>GTAACTGGTCGCGTCGAGGAACTACTGTCGCCAGGTGTCTCCCGCAGGGTGCGCCGGCAGCCTCGGATCCCGA<br>CTGGCGAGAGCCAGGTAACGAATGGATCCGAGGAACTACTGTCGGACCTGAAGGAGGCACGGGTCGACGGTACC<br>CAGTGGACGGGTATCGTCGCGGAGCTTCGGCTCCGGACCGCGTCCACTGCGGTCCGCACTCTGC
3H-AE-MG-spinach (without 120°KL)	416	AACTCTCGATCAGCGGACAGCGCTAATACGACTCACTATAGGGAGATACCTGGGAATGTGCTCTTCGGAGCATATT<br>CGGCCTGCTTCGGCAGGCGCTCGCTTCGGCGAGCGCAGGGTGAAGCCTCCACGCCCTGCCCGGCAGCCTCGAC<br>GCGACCGAAATGGTGAAGGACGGGTCCAGTGCTTCGGCACTGTTGAGTAGAGTGTGAGCTCCGTAACTGGTCGC<br>GTCGAGGAACTACTGTCGCCAGGTGTCTCCCGCAGGGTGCGCCGGCAGCCTCGGATCCCGACTGGCGAGAGCCA<br>GGTAACGAATGGATCCGAGGAACTACTGTCGGACCTGAAGGAGGCACGGGTCGACGGTACCCTTCGGGGTATCGT<br>CGCGGAGCTTCGGCTCCGGACCGCTTCGGCGGTCCGCACTCTGC
3H-AE-MG-spinach (with streptavidin aptamer) (without 120°KL)	430	AACTCTCGATCAGCGGACAGCGCTAATACGACTCACTATAGGGAGATACCTGGGAATGTGCTCTTCGGAGCATATT<br>CGGCCTGCTTCGGCAGGCGCTCGCTTCGGCGAGCGCAGGGTGAAGCCTCCACGCCCTGCCCGGCAGCCTCGAC<br>GCGACCGAAATGGTGAAGGACGGGTCCAGTGCTTCGGCACTGTTGAGTAGAGTGTGAGCTCCGTAACTGGTCGC<br>GTCGAGGAACTACTGTCGCCAGGTGTCTCCCGCAGGGTGCGCCGGCAGATGCGGCCGCCGACCAGAATCATGC<br>AAGTGCGTAAGATAGTCGCGGGTCGGCGGCCGCATAACTACTGTCGGACCTGAAGGAGGCACGGGTCGACGGT<br>ACCCTTCGGGGTATCGTCGCGGAGCTTCGGCTCCGGACCGCTTCGGCGGTCCGCACTCTGC
3H-AE-MG-Spinach (with PP7 aptamer) (without 120°KL)	403	AACTCTCGATCAGCGGACAGCGCTAATACGACTCACTATAGGGAGATACCTGGGAATGTGCTCTTCGGAGCATA<br>TTCGGCCTGCTTCGGCAGGCGCTCGCTTCGGCGAGCGCAGGGTGAAGCCTCCACGCCCTGCCCGGCAGCCTC<br>GACGCGACCGAAATGGTGAAGGACGGGTCCAGTGCTTCGGCACTGTTGAGTAGAGTGTGAGCTCCGTAACTGG<br>TCGCGTCGAGGAACTACTGTCGCCAGGTGTCTCCCGCAGGGTGCGCCGGCAGCCTCGGCACAGAAGATATGGC<br>TTCGTGCCGAGGAACTACTGTCGGACCTGAAGGAGGCACGGGTCGACGGTACCCTTCGGGGTATCGTCGCGGA<br>GCTTCGGCTCCGGACCGCTTCGGCGGTCCGCACTCTGC

Table S2. RNA sequences corresponding to the individual tiles & their lengths.

	Length (nt)	Sequence (5'-3')
3H-AE	248	GGGAGAUACCUGGGAAUGUGCUCACGUCUCGAGCAUAUUCGGCCUGCUUCGGCAGGCGCUCGCGAG<br>ACGUGCGAGCGCAGGGUGAAGCCUCCACGCCCUGCCCUCACUUCGGUGAGCCAGGUGUCUCCCGCA<br>GGGUGCGCGUGGCUUCGGCCACGACCUGAAGGAGGCACGGGUCGACGGUACCCAGUGGACGGGUAU<br>CGUCGCGGAGCUUCGGCUCCGGACCGCGUCCACUGCGGUCCGCACUCUGC
3H-AE-MG-V1	297	GGGAGAUACCUGGGAAUGUGCUCACGUCUCGAGCAUAUUCGGCCUGCUUCGGCAGGCGCUCGCGAG<br>ACGUGCGAGCGCAGGGUGAAGCCUCCACGCCCUGCCCUCACUUCGGUGAGCCAGGUGUCUCCCGCA<br>GGGUGCGCCGGCAGCCUCGGAUCCCGACUGGCGAGAGCCAGGUAACGAAUGGAUCCGAGGAACUACU<br>GUCGGACCUGAAGGAGGCACGGGUCGACGGUACCCAGUGGACGGGUAUCGUCGCGGAGCUUCGGCU<br>CCGGACCGCGUCCACUGCGGUCCGCACUCUGC
3H-AE-MG-V2	303	GGGAGAUACCUGGGAAUGUGCUCACGUCUCGAGCAUAUUCGGCCUGCUUCGGCAGGCGCUCGCGAGA<br>CGUGCGAGCGCAGGGUGAAGCCUCCACGCCCUGCCCUCACUUCGGUGAGCCAGGUGUCUCCCGCAGG<br>GUGCGCGCUGGCCCCUCCAGGAUCCCGACUGGCGAGAGCCAGGUAACGAAUGGAUCCUGUGAGGAACU<br>AGGCCGCGACCUGAAGGAGGCACGGGUCGACGGUACCCAGUGGACGGGUAUCGUCGCGGAGCUUCGGC<br>UCCGGACCGCGUCCACUGCGGUCCGCACUCUGC
3H-AE-MG-Spinach (with 120°KL)	388	GGGAGAUACCUGGGAAUGUGCUCACGUCUCGAGCAUAUUCGGCCUGCUUCGGCAGGCGCUCGCGAGA<br>CGUGCGAGCGCAGGGUGAAGCCUCCACGCCCUGCCCGGCAGCCUCGACGCGACCGAAAUGGUGAAGG<br>ACGGGUCCAGUGCUUCGGCACUGUUGAGUAGAGUGUGAGCUCCGUAACUGGUCGCGUCGAGGAACUA<br>CUGUCGCCAGGUGUCUCCCGCAGGGUGCGCCGGCAGCCUCGGAUCCCGACUGGCGAGAGCCAGGUAA<br>CGAAUGGAUCCGAGGAACUACUGUCGGACCUGAAGGAGGCACGGGUCGACGGUACCCAGUGGACGGGU<br>AUCGUCGCGGAGCUUCGGCUCCGGACCGCGUCCACUGCGGUCCGCACUCUGC
3H-AE-MG-Spinach (without 120°KL)	376	GGGAGAUACCUGGGAAUGUGCUCUUCGGAGCAUAUUCGGCCUGCUUCGGCAGGCGCUCGCUUCGGCG<br>AGCGCAGGGUGAAGCCUCCACGCCCUGCCCGGCAGCCUCGACGCGACCGAAAUGGUGAAGGACGGGUC<br>CAGUGCUUCGGCACUGUUGAGUAGAGUGUGAGCUCCGUAACUGGUCGCGUCGAGGAACUACUGUCGCC<br>AGGUGUCUCCCGCAGGGUGCGCCGGCAGCCUCGGAUCCCGACUGGCGAGAGCCAGGUAACGAAUGGAU<br>CCGAGGAACUACUGUCGGACCUGAAGGAGGCACGGGUCGACGGUACCCUUCGGGGUAUCGUCGCGGAG<br>CUUCGGCUCCGGACCGCUUCGGCGGUCCGCACUCUGC
3H-AE-MG-Spinach (with streptavidin aptamer)	390	GGGAGAUACCUGGGAAUGUGCUCUUCGGAGCAUAUUCGGCCUGCUUCGGCAGGCGCUCGCUUCGGCGA<br>GCGCAGGGUGAAGCCUCCACGCCCUGCCCGGCAGCCUCGACGCGACCGAAAUGGUGAAGGACGGGUCC<br>AGUGCUUCGGCACUGUUGAGUAGAGUGUGAGCUCCGUAACUGGUCGCGUCGAGGAACUACUGUCGCCA<br>GGUGUCUCCCGCAGGGUGCGCCGGCAGAUGCGGCCGCCGACCAGAAUCAUGCAAGUGCGUAAGAUAGU<br>CGCGGGUCGGCGGCCGCAUAACUACUGUCGGACCUGAAGGAGGCACGGGUCGACGGUACCCUUCGGGG<br>UAUCGUCGCGGAGCUUCGGCUCCGGACCGCUUCGGCGGUCCGCACUCUGC
3H-AE-MG-Spinach (with PP7 aptamer)	363	GGGAGAUACCUGGGAAUGUGCUCUUCGGAGCAUAUUCGGCCUGCUUCGGCAGGCGCUCGCUUCGGCGAG<br>CGCAGGGUGAAGCCUCCACGCCCUGCCCGGCAGCCUCGACGCGACCGAAAUGGUGAAGGACGGGUCCAG<br>UGCUUCGGCACUGUUGAGUAGAGUGUGAGCUCCGUAACUGGUCGCGUCGAGGAACUACUGUCGCCAGGU<br>GUCUCCCGCAGGGUGCGCCGGCAGCCUCGGCACAGAAGAUAUGGCUUCGUGCCGAGGAACUACUGUCGG<br>ACCUGAAGGAGGCACGGGUCGACGGUACCCUUCGGGGUAUCGUCGCGGAGCUUCGGCUCCGGACCGCUU<br>CGGCGGUCCGCACUCUGC

Table S3. Primer sequences for PCR of DNA gBlocks encoding RNA tiles.

	Length (nt)	Tm (°C)	GC (%)	Sequence (5'-3')
Forward Primer (FP) (for all RNA tiles)	22	72	59	AACTCTCGATCAGCGGACAGCG
Reverse Primer (RP) for all RNA tiles with 120 °KL	20	74	70	GCAGAGTGCGGACCGCAGTG
Reverse Primer (RP) for all RNA tiles without 120 °KL	20	79	70	GCAGAGTGCGGACCGCCGAA

Table S4. PCR reaction and cycle conditions for DNA gBlock amplification.

PCR reaction

	Stock	final	Volume (μl)
Forward Primer (FP)	10 μM	500 nM	2.5
Reverse Primer (RP)	10 μM	500 nM	2.5
DNA gBlock	10 ng/μl	50 ng	5
Phusion master mix (NEB)	2X	1X	25
nF water			15
Total			50

PCR cycle

	Steps
1	98°C for 30sec
2	98°C for 30sec
3	72°C for 30sec
4	Repeat steps 2-3 for 24 cycles
5	72°C for 10min
6	4°C forever, time: ~ 1 hour

Table S5. Primer sequences for Gibson Assembly.

	Length (nt)	Tm (°C)	GC (%)	Sequence (5'-3')
Insert (FP)	40	73.47	60	CGAACGATGAAAACTATGCGACGCGTGGTGGCGGCGGTTC
Insert (RP)	40	75.19	62.5	TGGTGGTGGTGCTCGAGTTAACGACCCAGCGGCACCAGGT
Vector (FP)	40	75.19	62.5	ACCTGGTGCCGCTGGGTCGTTAACTCGAGCACCACCACCA
Vector (RP)	40	73.47	60	GAACCGCCGCCACCACGCGTCGCATAGTTTTCATCGTTCG

Table S6. PCR reaction and cycle conditions for Gibson Assembly.

PCR Reaction

			Vector	Insert
	Stock	final	Volume (μl)	Volume (μl)
Forward Primer (FP)	10 μM	500 nM	3	3
Reverse Primer (RP)	10 μM	500 nM	3	3
DNA	10 ng/μl	50 ng	0.5	5 (50 ng)
Phusion master mix	2X	1X	30	30
nF water			23.5	19
Total			60	60

PCR cycle

Steps	Vector	Insert
1	98°C for 30sec	98°C for 30sec
2	98°C for 30sec	98°C for 30sec
3	60°C for 30sec	60°C for 30sec
4	72°C for 3min	72°C for 30sec
5	Repeat steps 2-4 for 10 cycles	Repeat steps 2-4 for 24 cycles
6	98°C for 30sec	72°C for 10min
5	72°C for 3min	4°C forever
6	Repeat step 7 for 20 cycles	
7	72°C for 10min	
8	4°C forever	

Table S7. mCherry-pET28 sequence, mCherry-pET28-linker-PCP sequence and linker-PCP sequences.

	Length (nt)	Sequence (5'-3')
mCherry-pET28b	6037	CCGTCTCCGGGAGCTGCATGTGTCAGAGGTTTTCACCGTCATCACCGAAACGCGCGAGGCAGCTGCGGTAAAGCTCA<br>TCAGCGTGGTCGTGAAGCGATTCACAGATGTCTGCCTGTTCATCCGCGTCCAGCTCGTTGAGTTTCTCCAGAAGCGTT<br>AATGTCTGGCTTCTGATAAAGCGGGCCATGTTAAGGGCGGTTTTTTCCTGTTTGGTCACTGATGCCTCCGTGTAAGGG<br>GGATTTCTGTTCATGGGGGTAATGATACCGATGAAACGAGAGAGGATGCTCACGATACGGGTTACTGATGATGAACAT<br>GCCCGGTTACTGGAACGTTGTGAGGGTAAACAACTGGCGGTATGGATGCGGCGGGACCAGAGAAAAATCACTCAGGG<br>TCAATGCCAGCGCTTCGTTAATACAGATGTAGGTGTTCCACAGGGTAGCCAGCAGCATCCTGCGATGCAGATCCGGAA<br>CATAATGGTGCAGGGCGCTGACTTCCGCGTTTCCAGACTTTACGAAACACGGAAACCGAAGACCATTCATGTTGTTGCT<br>CAGGTCGCAGACGTTTTGCAGCAGCAGTCGCTTCACGTTCGCTCGCGTATCGGTGATTCATTCTGCTAACCAGTAAGG<br>CAACCCCGCCAGCCTAGCCGGGTCCTCAACGACAGGAGCACGATCATGCGCACCCGTGGGGCCGCCATGCCGGCGA<br>TAATGGCCTGCTTCTCGCCGAAACGTTTGGTGGCGGGACCAGTGACGAAGGCTTGAGCGAGGGCGTGCAAGATTCCG<br>AATACCGCAAGCGACAGGCCGATCATCGTCGCGCTCCAGCGAAAGCGGTCCTCGCCGAAAATGACCCAGAGCGCTGC<br>CGGCACCTGTCCTACGAGTTGCATGATAAAGAAGACAGTCATAAGTGCGGCGACGATAGTCATGCCCCGCGCCCACCG<br>GAAGGAGCTGACTGGGTTGAAGGCTCTCAAGGGCATCGGTCGAGATCCCGGTGCCTAATGAGTGAGCTAACTTACATT<br>AATTGCGTTGCGCTCACTGCCCGCTTTCCAGTCGGGAAACCTGTCGTGCCAGCTGCATTAATGAATCGGCCAACGCGC<br>GGGGAGAGGCGGTTTGCGTATTGGGCGCCAGGGTGGTTTTTCTTTTCACCAGTGAGACGGGCAACAGCTGATTGCCC<br>TTCACCGCCTGGCCCTGAGAGAGTTGCAGCAAGCGGTCCACGCTGGTTTGCCCCAGCAGGCGAAAATCCTGTTTGATG<br>GTGGTTAACGGCGGGATATAACATGAGCTGTCTTCGGTATCGTCGTATCCCACTACCGAGATATCCGCACCAACGCGCA<br>GCCCGGACTCGGTAATGGCGCGCATTGCGCCCAGCGCCATCTGATCGTTGGCAACCAGCATCGCAGTGGGAACGATG<br>CCCTCATTCAGCATTTGCATGGTTTGTTGAAAACCGGACATGGCACTCCAGTCGCCTTCCCGTTCCGCTATCGGCTGAA<br>TTTGATTGCGAGTGAGATATTTATGCCAGCCAGCCAGACGCAGACGCGCCGAGACAGAACTTAATGGGCCCGCTAACAG<br>CGCGATTTGCTGGTGACCCAATGCGACCAGATGCTCCACGCCCAGTCGCGTACCGTCTTCATGGGAGAAAATAATACTG<br>TTGATGGGTGTCTGGTCAGAGACATCAAGAAATAACGCCGGAACATTAGTGCAGGCAGCTTCCACAGCAATGGCATCCT<br>GGTCATCCAGCGGATAGTTAATGATCAGCCCACTGACGCGTTGCGCGAGAAGATTGTGCACCGCCGCTTTACAGGCTTC<br>GACGCCGCTTCGTTCTACCATCGACACCACCACGCTGGCACCCAGTTGATCGGCGCGAGATTTAATCGCCGCGACAATT<br>TGCGACGGCGCGTGCAGGGCCAGACTGGAGGTGGCAACGCCAATCAGCAACGACTGTTTGCCCGCCAGTTGTTGTGCC<br>ACGCGGTTGGGAATGTAATTCAGCTCCGCCATCGCCGCTTCCACTTTTTCCCGCGTTTTCGCAGAAACGTGGCTGGCCTG<br>GTTCACCACGCGGGAAACGGTCTGATAAGAGACACCGGCATACTCTGCGACATCGTATAACGTTACTGGTTTCACATTCA<br>CCACCCTGAATTGACTCTCTTCCGGGCGCTATCATGCCATACCGCGAAAGGTTTTGCGCCATTCGATGGTGTCCGGGATC<br>TCGACGCTCTCCCTTATGCGACTCCTGCATTAGGAAGCAGCCCAGTAGTAGGTTGAGGCCGTTGAGCACCGCCGCCGCA<br>AGGAATGGTGCATGCAAGGAGATGGCGCCCAACAGTCCCCCGGCCACGGGGCCTGCCACCATACCCACGCCGAAACAA<br>GCGCTCATGAGCCCGAAGTGGCGAGCCCGATCTTCCCCATCGGTGATGTCGGCGATATAGGCGCCAGCAACCGCACCTG<br>TGGCGCCGGTGATGCCGGCCACGATGCGTCCGGCGTAGAGGATCGAGATCTCGATCCCGCGAAATTAATACGACTCACT<br>ATAGGGGAATTGTGAGCGGATAACAATTCCCCTCTAGAAATAATTTTGTTTAACTTTAAGAAGGAGATATACCATGGGCAGC<br>AGCCATCATCATCATCATCACAGCAGCGGCCTGGTGCCGCGCGGCAGCCATATGATGGTCAGCAAGGGCGAGGAGGATA<br>ACATGGCCATCATCAAGGAGTTCATGCGCTTCAAGGTGCACATGGAGGGCTCCGTGAACGGCCACGAGTTCGAGATCGAg<br>gGcGAGGGCGAGGGCCGCCCCTACGAGGGCACCCAGACCGCCAAGCTGAAGGTGACCAAGGGTGGCCCCCTGCCCTTCG<br>CCTGGGACATCCTGTCCCCTCAGTTCATGTACGGCTCCAAGGCCTACGTGAAGCAcCCCGCCGACATCCCCGACTACTTGA<br>AGCTGTCCTTCCCCGAGGGCTTCAAGTgGGAGCGCGTGATGAACTTCGAGGACGGCGGCGTGGTGACCGTGACCCAGGAC<br>TCCTCCCTGCAAGACGGCGAGTTCATCTACAAGGTGAAGCTGCGCGGCACCAACTTCCCCTCCGACGGCCCCGTAATGCA<br>GAAGAAGACTATGGGCTGGGAGGCCTCCTCCGAGCGGATGTACCCCGAGGACGGCGCCCTGAAGGGCGAGATCAAGCAG<br>AGGCTGAAGCTGAAGGACGGCGGCCACTACGACGCTGAGGTCAAGACCACCTACAAGGCCAAGAAGCCCGTGCAGCTGC<br>CCGGCGCCTACAACGTCAACATCAAGTTGGACATCACCTCCCACAACGAGGACTACACCATCGTGGAACAGTACGAACGCG<br>CCGAGGGCCGCCACTCCACCGGCGGCATGGACGAGCTGTACAAGCGCCCGGCGGCGAACGATGAAAACTATGCGTAACT<br>CGAGCACCACCACCACCACCACTGAGATCCGGCTGCTAACAAAGCCCGAAAGGAAGCTGAGTTGGCTGCTGCCACCGCT<br>GAGCAATAACTAGCATAACCCCTTGGGGCCTCTAAACGGGTCTTGAGGGGTTTTTTGCTGAAAGGAGGAACTATATCCGGA<br>TTGGCGAATGGGACGCGCCCTGTAGCGGCGCATTAAGCGCGGCGGGTGTGGTGGTTACGCGCAGCGTGACCGCTACACT<br>TGCCAGCGCCCTAGCGCCCGCTCCTTTCGCTTTCTTCCCTTCCTTTCTCGCCACGTTCGCCGGCTTTCCCCGTCAAGCTCT<br>AAATCGGGGGCTCCCTTTAGGGTTCCGATTTAGTGCTTTACGGCACCTCGACCCCAAAAAACTTGATTAGGGTGATGGTTC<br>ACGTAGTGGGCCATCGCCCTGATAGACGGTTTTTCGCCCTTTGACGTTGGAGTCCACGTTCTTTAATAGTGGACTCTTGTT<br>CCAAACTGGAACAACACTCAACCCTATCTCGGTCTATTCTTTTGATTTATAAGGGATTTTGCCGATTTCGGCCTATTGGTTA<br>AAAAATGAGCTGATTTAACAAAAATTTAACGCGAATTTTAACAAAATATTAACGTTTACAATTTCAGGTGGCACTTTTCGGGG<br>AAATGTGCGCGGAACCCCTATTTGTTTATTTTTCTAAATACATTCAAATATGTATCCGCTCATGAATTAATTCTTAGAAAAACT<br>CATCGAGCATCAAATGAAACTGCAATTTATTCATATCAGGATTATCAATACCATATTTTTGAAAAAGCCGTTTCTGTAATGAA<br>GGAGAAAACTCACCGAGGCAGTTCCATAGGATGGCAAGATCCTGGTATCGGTCTGCGATTCCGACTCGTCCAACATCAAT<br>ACAACCTATTAATTTCCCCTCGTCAAAAATAAGGTTATCAAGTGAGAAATCACCATGAGTGACGACTGAATCCGGTGAGAA<br>TGGCAAAAGTTTATGCATTTCTTTCCAGACTTGTTCAACAGGCCAGCCATTACGCTCGTCATCAAAATCACTCGCATCAAC<br>CAAACCGTTATTCATTCGTGATTGCGCCTGAGCGAGACGAAATACGCGATCGCTGTTAAAAGGACAATTACAAACAGGAA<br>TCGAATGCAACCGGCGCAGGAACACTGCCAGCGCATCAACAATATTTTCACCTGAATCAGGATATTCTTCTAATACCTGG<br>AATGCTGTTTTCCCGGGGATCGCAGTGGTGAGTAACCATGCATCATCAGGAGTACGGATAAAATGCTTGATGGTCGGAA<br>GAGGCATAAATTCCGTCAGCCAGTTTAGTCTGACCATCTCATCTGTAACATCATTGGCAACGCTACCTTTGCCATGTTTC<br>AGAAACAACTCTGGCGCATCGGGCTTCCCATACAATCGATAGATTGTCGCACCTGATTGCCCGACATTATCGCGAGCCC<br>ATTTATACCCATATAAATCAGCATCCATGTTGGAATTTAATCGCGGCCTAGAGCAAGACGTTTCCCGTTGAATATGGCTCA<br>TAACACCCCTTGTATTACTGTTTATGTAAGCAGACAGTTTTATTGTTCATGACCAAAATCCCTTAACGTGAGTTTTCGTTCC<br>ACTGAGCGTCAGACCCCGTAGAAAAGATCAAAGGATCTTCTTGAGATCCTTTTTTTCTGCGCGTAATCTGCTGCTTGCAAA<br>CAAAAAAACCACCGCTACCAGCGGTGGTTTGTTTGCCGGATCAAGAGCTACCAACTCTTTTTCCGAAGGTAACTGGCTTC<br>AGCAGAGCGCAGATACCAAATACTGTCCTTCTAGTGTAGCCGTAGTTAGGCCACCACTTCAAGAACTCTGTAGCACCGCC<br>TACATACCTCGCTCTGCTAATCCTGTTACCAGTGGCTGCTGCCAGTGGCGATAAGTCGTGTCTTACCGGGTTGGACTCAA<br>GACGATAGTTACCGGATAAGGCGCAGCGGTCGGGCTGAACGGGGGGTTCGTGCACACAGCCCAGCTTGGAGCGAACGA<br>CCTACACCGAACTGAGATACCTACAGCGTGAGCTATGAGAAAGCGCCACGCTTCCCGAAGGGAGAAAGGCGGACAGGTA<br>TCCGGTAAGCGGCAGGGTCGGAACAGGAGAGCGCACGAGGGAGCTTCCAGGGGGAAACGCCTGGTATCTTTATAGTCCT<br>GTCGGGTTTCGCCACCTCTGACTTGAGCGTCGATTTTTGTGATGCTCGTCAGGGGGGCGGAGCCTATGGAAAAACGCCAG<br>CAACGCGGCCTTTTTACGGTTCCTGGCCTTTTGCTGGCCTTTTGCTCACATGTTCTTTCCTGCGTTATCCCCTGATTCTGTG<br>GATAACCGTATTACCGCCTTTGAGTGAGCTGATACCGCTCGCCGCAGCCGAACGACCGAGCGCAGCGAGTCAGTGAG<br>CGAGGAAGCGGAAGAGCGCCTGATGCGGTATTTTCTCCTTACGCATCTGTGCGGTATTTCACACCGCATATATGGTGC<br>ACTCTCAGTACAATCTGCTCTGATGCCGCATAGTTAAGCCAGTATACACTCCGCTATCGCTACGTGACTGGGTCATGGC<br>TGCGCCCCGACACCCGCCAACACCCGCTGACGCGCCCTGACGGGCTTGTCTGCTCCCGGCATCCGCTTACAGACAAG<br>CTGTGA

mCherry-pET28b-linker-PCP 6424

CCGTCTCCGGGAGCTGCATGTGTCAGAGGTTTTCACCGTCATCACCGAAACGCGCGAGGCAGCTGCGGTAAAGCTCAT
CAGCGTGGTCGTGAAGCGATTCACAGATGTCTGCCTGTTCATCCGCGTCCAGCTCGTTGAGTTTCTCCAGAAGCGTTAA
TGTCTGGCTTCTGATAAAGCGGGCCATGTTAAGGGCGGTTTTTTCCTGTTTGGTCACTGATGCCTCCGTGTAAGGGGGA
TTTCTGTTCATGGGGGTAATGATACCGATGAAACGAGAGAGGATGCTCACGATACGGGTTACTGATGATGAACATGCCC
GGTTACTGGAACGTTGTGAGGGTAAACAACTGGCGGTATGGATGCGGCGGGACCAGAGAAAAATCACTCAGGGTCAAT
GCCAGCGCTTCGTTAATACAGATGTAGGTGTTCCACAGGGTAGCCAGCAGCATCCTGCGATGCAGATCCGGAACATAAT
GGTGCAGGGCGCTGACTTCCGCGTTTCCAGACTTTACGAAACACGGAAACCGAAGACCATTCATGTTGTTGCTCAGGTC
GCAGACGTTTTGCAGCAGCAGTCGCTTCACGTTCGCTCGCGTATCGGTGATTCATTCTGCTAACCAGTAAGGCAACCCC
GCCAGCCTAGCCGGGTCCTCAACGACAGGAGCACGATCATGCGCACCCGTGGGGCCGCCATGCCGGCGATAATGGCC
TGCTTCTCGCCGAAACGTTTGGTGGCGGGACCAGTGACGAAGGCTTGAGCGAGGGCGTGCAAGATTCCGAATACCGCA
AGCGACAGGCCGATCATCGTCGCGCTCCAGCGAAAGCGGTCCTCGCCGAAAATGACCCAGAGCGCTGCCGGCACCTG
TCCTACGAGTTGCATGATAAAGAAGACAGTCATAAGTGCGGCGACGATAGTCATGCCCCGCGCCCACCGGAAGGAGCT
GACTGGGTTGAAGGCTCTCAAGGGCATCGGTCGAGATCCCGGTGCCTAATGAGTGAGCTAACTTACATTAATTGCGTTG
CGCTCACTGCCCGCTTTCCAGTCGGGAAACCTGTCGTGCCAGCTGCATTAATGAATCGGCCAACGCGCGGGGAGAGGC
GGTTTGCGTATTGGGCGCCAGGGTGGTTTTTCTTTTCACCAGTGAGACGGGCAACAGCTGATTGCCCTTCACCGCCTGG
CCCTGAGAGAGTTGCAGCAAGCGGTCCACGCTGGTTTGCCCCAGCAGGCGAAAATCCTGTTTGATGGTGGTTAACGGC
GGGATATAACATGAGCTGTCTTCGGTATCGTCGTATCCCACTACCGAGATATCCGCACCAACGCGCAGCCCGGACTCG
GTAATGGCGCGCATTGCGCCCAGCGCCATCTGATCGTTGGCAACCAGCATCGCAGTGGGAACGATGCCCTCATTCAG
CATTTGCATGGTTTGTTGAAAACCGGACATGGCACTCCAGTCGCCTTCCCGTTCCGCTATCGGCTGAATTTGATTGCGA
GTGAGATATTTATGCCAGCCAGCCAGACGCAGACGCGCCGAGACAGAACTTAATGGGCCCGCTAACAGCGCGATTTGC
TGGTGACCCAATGCGACCAGATGCTCCACGCCCAGTCGCGTACCGTCTTCATGGGAGAAAATAATACTGTTGATGGGTG
TCTGGTCAGAGACATCAAGAAATAACGCCGGAACATTAGTGCAGGCAGCTTCCACAGCAATGGCATCCTGGTCATCCAG
CGGATAGTTAATGATCAGCCCACTGACGCGTTGCGCGAGAAGATTGTGCACCGCCGCTTTACAGGCTTCGACGCCGCTT
CGTTCTACCATCGACACCACCACGCTGGCACCCAGTTGATCGGCGCGAGATTTAATCGCCGCGACAATTTGCGACGGCG
CGTGCAGGGCCAGACTGGAGGTGGCAACGCCAATCAGCAACGACTGTTTGCCCGCCAGTTGTTGTGCCACGCGGTTGG
GAATGTAATTCAGCTCCGCCATCGCCGCTTCCACTTTTTCCCGCGTTTTCGCAGAAACGTGGCTGGCCTGGTTCACCACG
CGGGAAACGGTCTGATAAGAGACACCGGCATACTCTGCGACATCGTATAACGTTACTGGTTTCACATTCACCACCCTGAA
TTGACTCTCTTCCGGGCGCTATCATGCCATACCGCGAAAGGTTTTGCGCCATTCGATGGTGTCCGGGATCTCGACGCTCT
CCCTTATGCGACTCCTGCATTAGGAAGCAGCCCAGTAGTAGGTTGAGGCCGTTGAGCACCGCCGCCGCAAGGAATGGTG
CATGCAAGGAGATGGCGCCCAACAGTCCCCCGGCCACGGGGCCTGCCACCATACCCACGCCGAAACAAGCGCTCATGA
GCCCGAAGTGGCGAGCCCGATCTTCCCCATCGGTGATGTCGGCGATATAGGCGCCAGCAACCGCACCTGTGGCGCCGG
TGATGCCGGCCACGATGCGTCCGGCGTAGAGGATCGAGATCTCGATCCCGCGAAATTAATACGACTCACTATAGGGGAAT
TGTGAGCGGATAACAATTCCCCTCTAGAAATAATTTTGTTTAACTTTAAGAAGGAGATATACCATGGGCAGCAGCCATCATC
ATCATCATCACAGCAGCGGCCTGGTGCCGCGCGGCAGCCATATGATGGTCAGCAAGGGCGAGGAGGATAACATGGCCAT
CATCAAGGAGTTCATGCGCTTCAAGGTGCACATGGAGGGCTCCGTGAACGGCCACGAGTTCGAGATCGAggGcGAGGGCG
AGGGCCGCCCCTACGAGGGCACCCAGACCGCCAAGCTGAAGGTGACCAAGGGTGGCCCCCTGCCCTTCGCCTGGGACAT
CCTGTCCCCTCAGTTCATGTACGGCTCCAAGGCCTACGTGAAGCAcCCCGCCGACATCCCCGACTACTTGAAGCTGTCCTT
CCCCGAGGGCTTCAAGTgGGAGCGCGTGATGAACTTCGAGGACGGCGGCGTGGTGACCGTGACCCAGGACTCCTCCCTG
CAAGACGGCGAGTTCATCTACAAGGTGAAGCTGCGCGGCACCAACTTCCCCTCCGACGGCCCCGTAATGCAGAAGAAGAC
TATGGGCTGGGAGGCCTCCTCCGAGCGGATGTACCCCGAGGACGGCGCCCTGAAGGGCGAGATCAAGCAGAGGCTGAA
GCTGAAGGACGGCGGCCACTACGACGCTGAGGTCAAGACCACCTACAAGGCCAAGAAGCCCGTGCAGCTGCCCGGCGC
CTACAACGTCAACATCAAGTTGGACATCACCTCCCACAACGAGGACTACACCATCGTGGAACAGTACGAACGCGCCGAGG
GCCGCCACTCCACCGGCGGCATGGACGAGCTGTACAAGCGCCCGGCGGCGAACGATGAAAACTATGCGACGCGTGGTG
GCGGCGGTTCGATGAGCAAGACTATCGTTTTGTCCGTCGGCGAGGCTACCCGTACCTTGACCGAAATTCAATCCACCGCG
GACCGTCAAATTTTTGAGGAAAAAGTCGGTCCTCTGGTGGGTCGTCTGCGTCTGACCGCGAGCCTGCGCCAGAACGGTG
CCAAAACGGCATACCGTGTTAATCTGAAACTGGATCAGGCCGACGTTGTGGACAGCGGTCTGCCGAAAGTCCGCTACAC
CCAGGTGTGGAGCCACGATGTGACGATCGTTGCGAATAGCACCGAAGCGAGCCGCAAGAGCCTGTACGACCTGACCAA
GAGCCTGGTGGCAACGTCCCAAGTTGAAGATCTGGTTGTTAACCTGGTGCCGCTGGGTCGTTAACTCGAGCACCACCACC
ACCACCACTGAGATCCGGCTGCTAACAAAGCCCGAAAGGAAGCTGAGTTGGCTGCTGCCACCGCTGAGCAATAACTAGCA
TAACCCCTTGGGGCCTCTAAACGGGTCTTGAGGGGTTTTTTGCTGAAAGGAGGAACTATATCCGGATTGGCGAATGGGACG
CGCCCTGTAGCGGCGCATTAAGCGCGGCGGGTGTGGTGGTTACGCGCAGCGTGACCGCTACACTTGCCAGCGCCCTAGC
GCCCGCTCCTTTCGCTTTCTTCCCTTCCTTTCTCGCCACGTTCGCCGGCTTTCCCCGTCAAGCTCTAAATCGGGGGCTCCC
TTTAGGGTTCCGATTTAGTGCTTTACGGCACCTCGACCCCAAAAAACTTGATTAGGGTGATGGTTCACGTAGTGGGCCATC
GCCCTGATAGACGGTTTTTCGCCCTTTGACGTTGGAGTCCACGTTCTTTAATAGTGGACTCTTGTTCCAAACTGGAACAAC
ACTCAACCCTATCTCGGTCTATTCTTTTGATTTATAAGGGATTTTGCCGATTTCGGCCTATTGGTTAAAAAATGAGCTGATTT
AACAAAAATTTAACGCGAATTTTAACAAAATATTAACGTTTACAATTTCAGGTGGCACTTTTCGGGGAAATGTGCGCGGAAC
CCCTATTTGTTTATTTTTCTAAATACATTCAAATATGTATCCGCTCATGAATTAATTCTTAGAAAAACTCATCGAGCATCAAATG
AAACTGCAATTTATTCATATCAGGATTATCAATACCATATTTTTGAAAAAGCCGTTTCTGTAATGAAGGAGAAAACTCACCGA
GGCAGTTCCATAGGATGGCAAGATCCTGGTATCGGTCTGCGATTCCGACTCGTCCAACATCAATACAACCTATTAATTTCCC
CTCGTCAAAAATAAGGTTATCAAGTGAGAAATCACCATGAGTGACGACTGAATCCGGTGAGAATGGCAAAAGTTTATGCATT
TCTTTCCAGACTTGTTCAACAGGCCAGCCATTACGCTCGTCATCAAAATCACTCGCATCAACCAAACCGTTATTCATTCGTGA
TTGCGCCTGAGCGAGACGAAATACGCGATCGCTGTTAAAAGGACAATTACAAACAGGAATCGAATGCAACCGGCGCAGGA
ACACTGCCAGCGCATCAACAATATTTTCACCTGAATCAGGATATTCTTCTAATACCTGGAATGCTGTTTTCCCGGGGATCGC
AGTGGTGAGTAACCATGCATCATCAGGAGTACGGATAAAATGCTTGATGGTCGGAAGAGGCATAAATTCCGTCAGCCAGTT
TAGTCTGACCATCTCATCTGTAACATCATTGGCAACGCTACCTTTGCCATGTTTCAGAAACAACTCTGGCGCATCGGGCTTC
CCATACAATCGATAGATTGTCGCACCTGATTGCCCGACATTATCGCGAGCCCATTTATACCCATATAAATCAGCATCCATGT
TGGAATTTAATCGCGGCCTAGAGCAAGACGTTTCCCGTTGAATATGGCTCATAACACCCCTTGTATTACTGTTTATGTAAGC
AGACAGTTTTATTGTTCATGACCAAAATCCCTTAACGTGAGTTTTCGTTCCACTGAGCGTCAGACCCCGTAGAAAAGATCAA
AGGATCTTCTTGAGATCCTTTTTTTCTGCGCGTAATCTGCTGCTTGCAAACAAAAAAACCACCGCTACCAGCGGTGGTTTGT
TTGCCGGATCAAGAGCTACCAACTCTTTTTCCGAAGGTAACTGGCTTCAGCAGAGCGCAGATACCAAATACTGTCCTTCTAG
TGTAGCCGTAGTTAGGCCACCACTTCAAGAACTCTGTAGCACCGCCTACATACCTCGCTCTGCTAATCCTGTTACCAGTG
GCTGCTGCCAGTGGCGATAAGTCGTGTCTTACCGGGTTGGACTCAAGACGATAGTTACCGGATAAGGCGCAGCGGTCG
GGCTGAACGGGGGGTTCGTGCACACAGCCCAGCTTGGAGCGAACGACCTACACCGAACTGAGATACCTACAGCGTGAG
CTATGAGAAAGCGCCACGCTTCCCGAAGGGAGAAAGGCGGACAGGTATCCGGTAAGCGGCAGGGTCGGAACAGGAGAG
CGCACGAGGGAGCTTCCAGGGGGAAACGCCTGGTATCTTTATAGTCCTGTCGGGTTTCGCCACCTCTGACTTGAGCGTC
GATTTTTGTGATGCTCGTCAGGGGGGCGGAGCCTATGGAAAAACGCCAGCAACGCGGCCTTTTTACGGTTCCTGGCCTTT
TGCTGGCCTTTTGCTCACATGTTCTTTCCTGCGTTATCCCCTGATTCTGTGGATAACCGTATTACCGCCTTTGAGTGAGCTG

ATACCGCTCGCCGCAGCCGAACGACCGAGCGCAGCGAGTCAGTGAGCGAGGAAGCGGAAGAGCGCCTGATGCGGTATTT
TCTCCTTACGCATCTGTGCGGTATTTCACACCGCATATATGGTGCACTCTCAGTACAATCTGCTCTGATGCCGCATAGTTAA
GCCAGTATACACTCCGCTATCGCTACGTGACTGGGTCATGGCTGCGCCCCGACACCCGCCAACACCCGCTGACGCGCCC
TGACGGGCTTGTCTGCTCCCGGCATCCGCTTACAGACAAGCTGTGA

Linker-PCP 390

ACGCGTGGTGGCGGCGGTTCGATGAGCAAGACTATCGTTTTGTCCGTCGGCGAGGCTACCCGTACCTTGACCGAAATT
CAATCCACCGCGGACCGTCAAATTTTTGAGGAAAAAGTCGGTCCTCTGGTGGGTCGTCTGCGTCTGACCGCGAGCCTG
CGCCAGAACGGTGCCAAAACGGCATACCGTGTTAATCTGAAACTGGATCAGGCCGACGTTGTGGACAGCGGTCTGCCG
AAAGTCCGCTACACCCAGGTGTGGAGCCACGATGTGACGATCGTTGCGAATAGCACCGAAGCGAGCCGCAAGAGCCTG
TACGACCTGACCAAGAGCCTGGTGGCAACGTCCCAAGTTGAAGATCTGGTTGTTAACCTGGTGCCGCTGGGTCGTTAA

References

1. Schindelin, J.; Arganda-Carreras, I.; Frise, E.; Kaynig, V.; Longair, M.; Pietzsch, T.; Preibisch, S.; Rueden, C.; Saalfeld, S.; Schmid, B.; Tinevez, J.-Y.; et al. Fiji: An open-source platform for biological-image analysis. *Nat. Methods* **2012**, *9*, 676–682, doi:10.1038/nmeth.2019.
2. Babendure, J.R.; Adams, S.R.; Tsien, R.Y. Aptamers switch on fluorescence of triphenylmethane dyes. *J. Am. Chem. Soc.* **2003**, *125*, 14716–14717, doi:10.1021/ja037994o.
3. Gasteiger, E.; Hoogland, C.; Gattiker, A.; Wilkins, M.R.; Appel, R.D.; Bairoch, A. Protein identification and analysis tools on the ExPASy server. In *The proteomics Protocols Handbook*, Springer: Berlin, Germany, 2005; pp. 571–607.
